# Complete chloroplast genome data reveal the existence of the *Solidago canadensis* L. complex and its potential introduction pathways into China

**DOI:** 10.3389/fpls.2024.1498543

**Published:** 2024-12-20

**Authors:** Yu-Tian Tao, Lu-Xi Chen, Ming Jiang, Jie Jin, Zhong-Shuai Sun, Chao-Nan Cai, Han-Yang Lin, Allison Kwok, Jun-Min Li, Mark van Kleunen

**Affiliations:** ^1^ Zhejiang Provincial Key Laboratory of Plant Evolutionary Ecology and Conservation, Taizhou University, Taizhou, China; ^2^ School of Electronics and Information Engineering, Taizhou University, Taizhou, China; ^3^ School of Advances Study, Taizhou University, Taizhou, China; ^4^ Environmental and Life Sciences Graduate Program, Trent University, Peterborough, ON, Canada; ^5^ Department of Biology, University of Konstanz, Konstanz, Germany

**Keywords:** *Solidago*, chloroplast genome, phylogenetic analysis, introduction pathways, molecular marker

## Abstract

*Solidago canadensis*, native to North America, is an invasive species in many areas of the world, where it causes serious damage to natural ecosystems and economic losses. However, a dearth of genetic resources and molecular markers has hampered our understanding of its invasion history. Here, we *de novo* assembled 40 complete chloroplast genomes of *Solidago* species, including 21 *S. canadensis* individuals, 15 *S. altissima* individuals, and four *S. decurrens* individuals, the sizes of which ranged from 152,412 bp to 153,170 bp. The phylogenetic trees based on the complete chloroplast genome sequences and nuclear genome-wide SNP data showed that *S. canadensis* and *S. altissima* cluster together and form a monophyletic pair, as sister to *S. decurrens*, indicating the existence of the *S. canadensis* L. complex in China. Three potential introduction pathways were identified. The chloroplast-genome structure and gene contents are conservative in the genomes of the *S. canadensis* L. complex and *S. decurrens*. The analysis of sequence divergence indicated five variable regions, and 10 chloroplast protein-coding genes that underwent positive selection were identified. Our findings shed new light on the invasion history of *S. canadensis* and the data sets generated in this study will facilitate future research on its chloroplast genome evolution.

## 1 Introduction

The genus *Solidago* L. (Asteraceae) has currently ca. 140 recognized species (Semple and Cook, 2006; Beck and Semple, 2015). The majority of *Solidago* species are native to North America, while a few species are native to South America or Eurasia (Beaudry and Chabot, 1957; McNeil, 1976). However, at least 50 of the North American *Solidago* species have been introduced as ornamental plants to Europe in the 17th and 18th century (Loudon, 1850), and most likely also elsewhere. Some of those species have become naturalized in Europe, as well as in Asia and other parts of the world (Weber, 2017; Van Kleunen et al., 2019). One of them, *Solidago canadensis* L., is now one of the most aggressive invasive plant species in Europe and in China (Xu et al., 2012; Zhao et al., 2015). However, due to differences in morphology and ploidy, there is uncertainty about whether the plants in China and Europe belong to the same species, and thus whether they both belong to *S. canadensis*.

The taxonomy of *S. canadensis* is complicated, and due to its wide variability in leaf sizes, pubescence, rhizome lengths, flowerhead sizes, and ploidy levels, multiple varieties are recognized (Croat, 1972; Melville and Morton, 1982; Beck and Semple, 2015). Some of the taxa that some taxonomists consider to be varieties of *S. canadensis* are considered to be separate species by others. Consequently, uncertainty exists around the true identity of what is now usually called *Solidago canadensis* in the invaded ranges. For example, a plant-morphological study by Weber (1997) concluded that the taxon in Europe is highly variable (Weber, 1997), and that, particularly due to its characteristic nodding shoot tips, it has to be closely related to *S. canadensis* var. *scabra* (Muhl.) Torr. & Gray, which is considered a synonym of *S. altissima* L (Fernald, 1950; Croat, 1972). Consequently, many older publications refer to this invasive species in Europe as *S. altissima* (Weber and Schmid, 1998). However, *S. altissima* is hexaploid, whereas the taxon in Europe is diploid. Since the early 2000s, most studies use the name *S. canadensis* (Van Kleunen and Schmid, 2003). However, Verloove et al. (2017) reported the first evidence for an established hexaploid population of *S. altissima* in Europe (Verloove et al., 2017). In Asia, a highly invasive hexaploid *Solidago* has been identified in Japan as *S. altissima* (https://www.nies.go.jp/biodiversity/invasive/DB/detail/80600e.html), but *S. canadensis* has also been identified as naturalized in Japan. In China, however, an highly invasive hexaploid *Solidago* has been identified as *S. canadensis*, but *S. altissima* has also been recorded as naturalized in China (Ma and Qiang, 2007). So, both in Europe and Asia, *S. altissima* and *S. canadensis* have been identified, but doubts about the exact taxonomic status remain.

Based on botanical identification literature, *S. altissima* should distinguish itself from *S. canadensis* by having fewer leaf serrations, more disk florets, longer ray florets, a higher involucral height, larger pollen, and a nodding shoot tip, and by being hexaploid instead of diploid (Croat, 1972; Melville and Morton, 1982; Weber, 1997). Based on an extensive comparison of morphological characteristics, Semple et al. (2015) concluded that *S. altissima* and *S. canadensis* should be treated as separate species (Semple et al., 2015). However, some plants, like *S. canadensis* in Europe, have characteristics of both species, and therefore molecular marker data might give more precise information on its identity (Weber, 1997; Semple et al., 2023). Recently, Tian et al. (2023) used nuclear ribosomal ITS and chroloplast trnH-psbA intergenic spacer sequences on specimens from North America, Europe and China, and found that specimens morphologically identified to be *S. canadensis* and *S. altissima* were >99% identical, and should be considered as one species complex (Tian et al., 2023). However, other molecular markers might provide more conclusive evidence.

Here, we used next-generation sequencing to obtain complete chloroplast genome sequences of 40 *Solidago* individuals from the native range in the US, and the non-native ranges in Asia (China and Japan) and Europe. An advantage of the chloroplast genome, compared to the nuclear genome, is that it is smaller and has more copy numbers (Ashworth, 2017). Furthermore, the chloroplast genome is usually maternally inherited and does not undergo gene recombination (Wang et al., 2023). Therefore, complete chloroplast genomes have frequently been used to study population genetics and species evolution, and to determine phylogenetic relationships among plant species (Zhang and Chen, 2022; Jiang et al., 2023). The aims of our study were to (i) obtain complete chloroplast genomes of specimens morphologically identified to be *S. canadensis* and *S. altissima*, to evaluate the usefulness of the chloroplast genome in species identification and phylogenetics, and (ii) to obtain insight into whether the invasive *Solidago* in Europe and Asia are most likely *S. canadensis* or *S. altissima*. Furthermore, we searched for highly variable genome regions that could be used to develop barcode markers, and we tested for genes with positive selection to identify genes that may play a role in evolutionary adaptation.

## 2 Materials and methods

### 2.1 Plant materials and genome data sources

For *S. canadensis* and *S. altissima*, seeds and leaf samples of a total of 36 individuals were collected from the native range (11 from the United States and 10 from Canada) and the introduced range (10 from China, 4 from Europe, and 1 from Japan). In detail, seeds of reference native *S. canadensis* L. and *S. altissima* L. (Asteraceae) were obtained from The Germplasm Resources Information Network of the United States Department of Agriculture (GRIN/USDA) with accession numbers W6 52837 and W6 57335, respectively. The seeds were sown and leaf material of one offspring plant from each of the *S. canadenisis* and *S. altissima* accessions were used for chloroplast-genomic analysis. Additionally, leaf samples of *S. altissima* herbarium specimens were obtained: three samples from the Wuhan Institute of Botany (HIB; accession numbers HIB0188587, HIB0188588, and HIB0188589) and one sample from the Chinese Field Herbarium (CFH; accession number CSH0115776). We also obtained leaf material of one *S. canadensis* specimen from the Specimen Museum of Zhejiang University (accession number HZU60102855; **Supplementary Table S1**). We also used 29 leaf samples of *S. canadensis* collected in the field, including nine samples from the United States, six samples from China, and four samples from Europe. In addition, we used 10 samples of *S. altissima* from Peterborough, Canada. The healthy leaf samples were washed and then dried in silica gel for preservation until DNA extraction. In total, we had 21 *S. canadensis* samples and 15 *S. altissima* samples. For the samples collected in the field, *S. canadensis* and *S. altissima* were mainly distinguished by the length and arrangement of epidermal hairs on stems and leaves, and the shape and margins of leaves. One individual each of *S. canadensis*, *S. altissima* and *S. decurrens* are shown in **Supplementary Figure S1**.

As a control to show that the chloroplast genomic analysis can distinguish *Solidago* species, we also used samples of *S. decurrens*, which is native in China. We had one *S. decurrens* herbarium specimen from the Specimen Museum of Zhejiang University (accession number HZU60120963), and three leaf samples of *S. decurrens* collected in the field in China (**Supplementary Tables S1, S2**). In addition, we also obtained complete chloroplast genome sequence data of *S. decurrens* (NC_053705.1) from the National Center for Biotechnology Information (NCBI, https://www.ncbi.nlm.nih.gov/). As additional outgroups, we also obtained from NCBI complete chloroplast genome sequence data of six other Asteraceae species, including *Aster flaccidus* (MN122101.1) (Zhou et al., 2019), *Conyza bonariensis* (NC_035884.1) (Hereward et al., 2017), *Erigeron canadensis* (MT806101.1), *Heteroplexis incana* (NC_048508.1), *Lagenophora cuchumatanica* (NC_034819.1) (Vargas et al., 2017), and *Symphyotrichum subulatum* (NC_050667.1) (Hu, 2020).

### 2.2 Total DNA extraction

Approximately 1 g of each of the dried leaf samples was used for genomic DNA extraction with the modified CTAB method (Doyle and Doyle, 1987). A library with insertion sizes of 300-500 bp was constructed for paired-end sequencing using the Illumina Novaseq 6000 platform (Illumina, San Diego, CA, United States).

### 2.3 Chloroplast genome assembly and annotation

Chloroplast genomes were *de novo* assembled using NOVOPlasty version 4.2 with default parameters (Dierckxsens et al., 2017). The GeSeq tool was used to annotate the assembled chloroplast genome sequences (Tillich et al., 2017). Results of software based annotation were verified by manual inspection using Geneious Prime 2021.1.1 (Biomatters Ltd., Auckland, New Zealand). Chloroplast gene circular maps were drawn with the OrganellarGenomeDRAW tool (OGDRAW) (Greiner et al., 2019).

### 2.4 Nuclear genome SNP data calling

For the nuclear phylogenetic tree construction, genome-wide SNP data was obtained according to the following steps. First, the raw sequencing data were cleaned using Trimmomatic (version 0.39) (Bolger et al., 2014) to remove adapters and low-quality sequences (the reads were scanned with a 4-base wide sliding window, cut when the average quality per base dropped below 30, and dropped when they were less than 120 bases long). Then the cleaned reads were mapped to the nuclear genome of *S. canadensis* using the software Burrows–Wheeler Alignment (BWA, version 0.7.17) with default parameters (Li and Durbin, 2009). The variant calling was performed using the Genome Analysis Toolkit (GATK) Best Practices Pipeline (version 4.2.0.0) (Van der Auwera et al., 2013), and variants were filtered with the VariantFiltration tool from GATK (-Window 4, -filter “QD < 2.0 || MQ<40.0 || DP <10 || FS > 60.0). Finally, the filtered SNPs of the *Solidago* species were merged using the software BCFtools (version 1.11) with default parameters (Danecek et al., 2021).

### 2.5 Phylogenetic tree construction

In the phylogenetic tree construction, the six non-*Solidago* species that belong to the same Asteraceae Tribe (*A. flaccidus*, *C. bonariensis*, *E. canadensis*, *H. incana*, *L. cuchumatanica*, and *S. subulatum*), served as outgroups for the chloroplast genome tree. In the nuclear genome tree, *S. subulatum* was served as outgroup. The whole-chloroplast-genome sequences were aligned using MAFFT v7.471 (Katoh and Standley, 2013) and manually adjusted. To determine the phylogenetic relationships, a maximum-likelihood tree of the chloroplast genome was constructed based on the manually adjusted aligned chloroplast genomes. The nuclear genome tree was built based on the genome-wide SNP data. The best-fit substitution model was selected by the software MrModeltest v2.4, which selects general time-reversible models with invariant sites and the gamma-rate heterogeneity (GTR+G+I) as the best-fitting substitution model. The maximum likelihood tree was constructed using IQ-TREE version 1.6.12 (Nguyen et al., 2015) with 1000 bootstrap replicates.

For principal component analysis (PCA), eigenvalues for consensus genotyping from SNP data of chloroplast genomes in *Solidago* species were calculated in plink v1.9 (Purcell et al., 2007). Then PCA plots were created by plotting the first two eigenvalues by ggplot2 package (Wickham, 2016) in R.

### 2.6 Comparative genome analysis

Chloroplast genome comparisons were carried out and visualized with the online software mVISTA (Frazer et al., 2004) based on annotation information of the sample *Solidago canadensis* 21 (**Supplementary Table S2**) in Shuffle LAGAN mode. DNAsp v5.10 software (Librado and Rozas, 2009) was used to perform nucleotide diversity (Pi) and sequence-polymorphism analysis, and the settings were a step size of 200 bp and a window length of 600 bp.

Positive selection analysis was performed by the software EasyCodeML (Gao et al., 2019) across the chloroplast genomes, using a site-specific model with five site models (M0, M1a & M2a, M7 & M8) to calculate the synonymous (dS) and non-synonymous (dN) substitution rates, and their ratio (ω = dN/dS). EasyCodeML uses CodeML of the PAML software (Gao et al., 2019). The site-specific model allowed the ω ratio to vary among sites while maintaining a fixed ω ratio in all the branches (Jiang et al., 2023). Specifically, M1a (nearly neutral) vs M2a (positive selection), and M7 (β) vs M8 (β & ω) were applied to find sequences that had undergone positive selection (Yang and Nielsen, 2002). Likelihood ratio tests (LRT) were used for selection-strength evaluation with the comparison of M1a vs M2a and M7 vs M8. A p value of the chi-square statistic smaller than 0.05 was considered to be significant. The site models of M2a and M8 were implemented using Bayes Empirical Bayes (BEB) inference (Yang et al., 2005) in order to estimate the posterior probabilities and positive selection pressures on the selected genes.

## 3 Results

### 3.1 Chloroplast-genome features of *S. canadensis*, *S. altissima*, and *S. decurrens*

**Figure 1:**
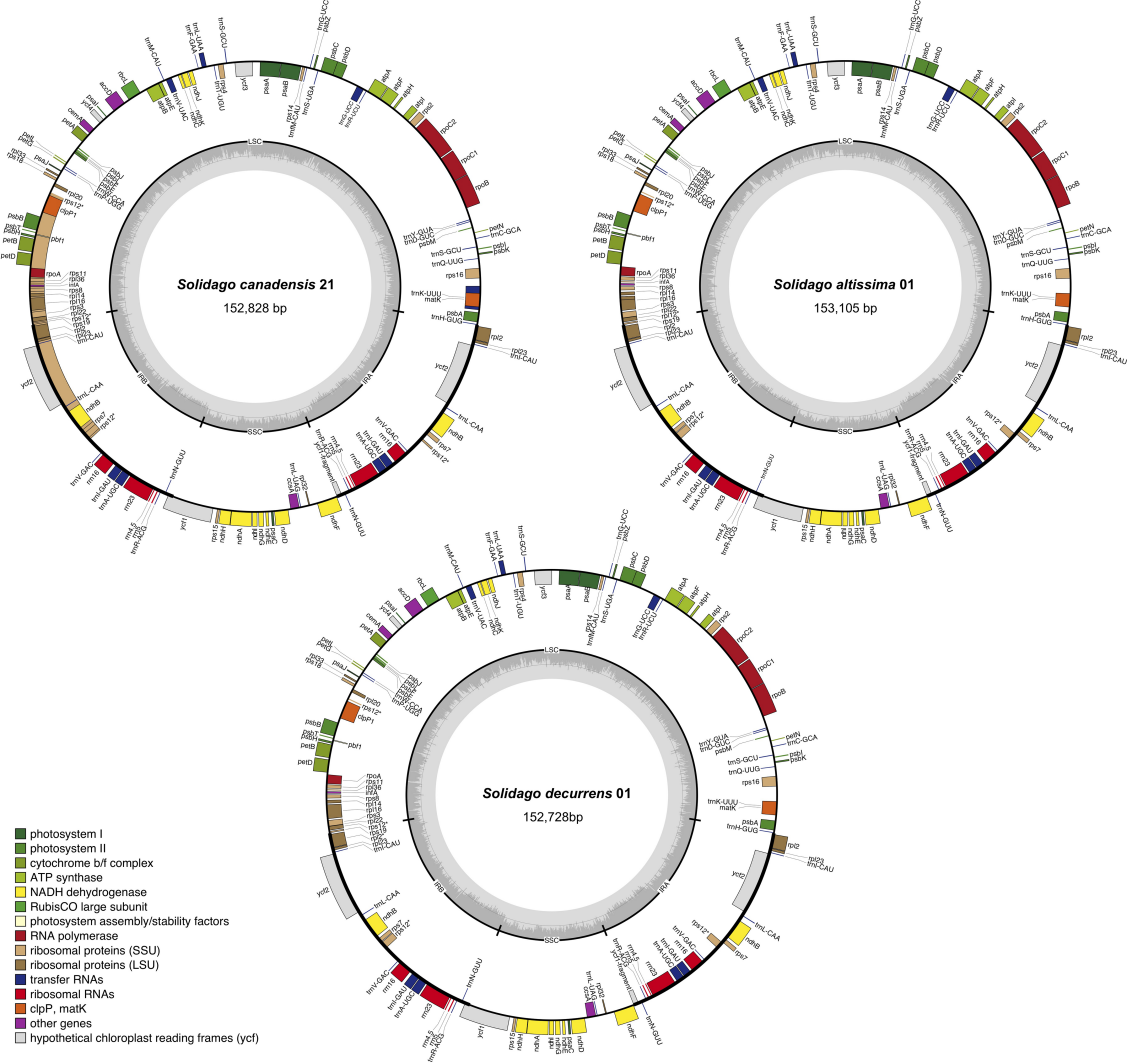
Gene map of the chloroplast genomes of *Solidago canadensis*, *S. altissima*, and *S. decurrens*. Genes that are located inside the circle were transcribed in the clockwise direction, and those located outside the circle were transcribed in the counter-clockwise direction. Genes belonging to different functional groups were marked in different colors. The dark-gray columns in the inner circle were related to the GC content, and the lighter-gray columns to the AT content.

There was a large overlap in the lengths of the complete circular chloroplast genomes of *S. canadensis* (152,412 to 153,170 bp; n = 21), *S. altissima* (152,957 to 153,126 bp; n = 15) and *S. decurrens* (152,728 to 152,863 bp; n = 5). Each chloroplast genome had a typical quadripartite structure, including a large single-copy region (LSC, 84,214 bp to 85,061 bp, 84,809 bp to 85,026 bp, and 84,599 bp to 84,736 bp, respectively for *S. canadensis*, *S. altissima*, and *S. decurrens*) and a small single-copy region (SSC, 18,066 bp to 18,143 bp, 18,066 bp to 18,165 bp, and 18,089 bp to 18,095 bp, respectively, for *S. canadensis*, *S. altissima*, and *S. decurrens*), separated by two inverted repeats (IRA and IRB, 25,015 bp to 25,134 bp, 25013 bp to 25024 bp, and 25,019 bp to 25,020 bp, respectively for *S. canadensis*, *S. altissima*, and *S. decurrens*) (**Supplementary Tables S1, S2** and **Figure 1**).

The chloroplast-genome features of the sampled *S. canadensis*, *S. altissima* and *S. decurrens* plants were largely identical. All the chloroplast genomes of *S. canadensis*, *S. altissima*, and *S. decurrens* showed similar GC contents ranging from 37.16% to 37.29%. The gene numbers and gene orders of the chloroplast genomes were identical. They contained a total of 128 genes, including 85 protein-coding genes, 35 tRNA genes, and 8 rRNA genes (**Supplementary Table S2**). Of the 128 genes, six protein-coding genes (*rpl2*, *rpl23*, *ycf2*, *ndhB*, *rps7*, and *ycf1*), seven tRNA genes (*trnI*-*CAU*, *trnL*-*CAA*, *trnV*-*GAC*, *trnI*-*GAU*, *trnA*-*UGC*, *trnR*-*ACG*, and *trnN*-*GUU*), and 4 rRNA genes (*rrn16*, *rrn23*, *rrn4.5*, and *rrn5*) were duplicated in the IR regions. The majority of encoded chloroplast genes are involved in photosynthesis-related metabolic processes (**Table 1**). There were 15 genes harboring a single intron (*trnK*-*UUU*, *rps16*, *rpoC1*, *atpF*, *trnG*-*UCC*, *trnL*-*UAA*, *trnV*-*UAC*, *petB*, *petD*, *rpl16*, *rpl2*, *ndhB*, *trnI*-*GAU*, *trnA*-*UGC*, and *ndhA*), and tree genes containing two introns (*ycf3*, *rps12*, and *clpP1*) (**Supplementary Table S3**).

**Table 1:** List of annotated genes in the chloroplast genome of *S. canadensis* L. complex and *S. decurrens*.

Group of genes	Gene names
Photosystem I	*psaA*, *psaB*, *psaC*, *psaI*, *psaJ*
Photosystem II	*psbA*, *psbB*, *psbC*, *psbD*, *psbE*, *psbF*, *psbH*, *psbI*, *psbJ*, *psbK*, *psbL*, *psbM*, *psbT*, *psbZ*, *pbf1*
Cytochrome b/f complex	*petA*, *petB*, *petD*, *petG*, *petL*, *petN*
ATP synthase	*atpA*, *atpB*, *atpE*, *atpF*, *atpH*, *atpI*
NADP dehydrogenase	*ndhA*, *ndhB*, *ndhC*, *ndhD*, *ndhE*, *ndhF*, *ndhG*, *ndhH*, *ndhI*, *ndhJ*, *ndhK*
RubisCO large subunit	*rbcL*
RNA polymerase	*rpoA*, *rpoB*, *rpoC1*, *rpoC2*
Ribosomal proteins (SSU)	*rps2*, *rps3*, *rps4*, *rps7*, *rps8*, *rps11*, *rps12*, *rps14*, *rps15*, *rps16*, *rps18*, *rps19*
Ribosomal proteins (LSU)	*rpl2*, *rpl14*, *rpl16*, *rpl20*, *rpl22*, *rpl23*, *rpl32*, *rpl33*, *rpl36*
Hypothetical chloroplast reading frames (ycf)	*ycf1*, *ycf2*, *ycf3*, *ycf4*
Other genes	*accD*, *ccsA*, *cemA*, *clpP1*, *infA*, *matK*
Ribosomal RNAs	*rrn16*, *rrn23*, *rrn4.5*, *rrn5*
Transfer RNAs	*trnH-GUG*, *trnK-UUU*, *trnQ-UUG*, *trnS-GCU*, *trnC-GCA*, *trnD-GUC*, *trnY-GUA*, *trnR-UCU*, *trnG-UCC*, *trnS-UGA*, *trnG-UCC*, *trnfM-CAU*, *trnS-GCU*, *trnT-UGU*, *trnL-UAA*, *trnF-GAA*, *trnV-UAC*, *trnM-CAU*, *trnW-CCA*, *trnP-UGG*, *trnI-CAU*, *trnL-CAA*, *trnV-GAC*, *trnI-GAU*, *trnA-UGC, trnR-ACG*, *trnN-GUU*, *trnL-UAG*, *trnN-GUU*, *trnR-ACG*, *trnA-UGC*, *trnI-GAU*, *trnV-GAC*, *trnL-CAA*, *trnI-CAU*

### 3.2 Phylogenetic analyses

**Figure 2:**
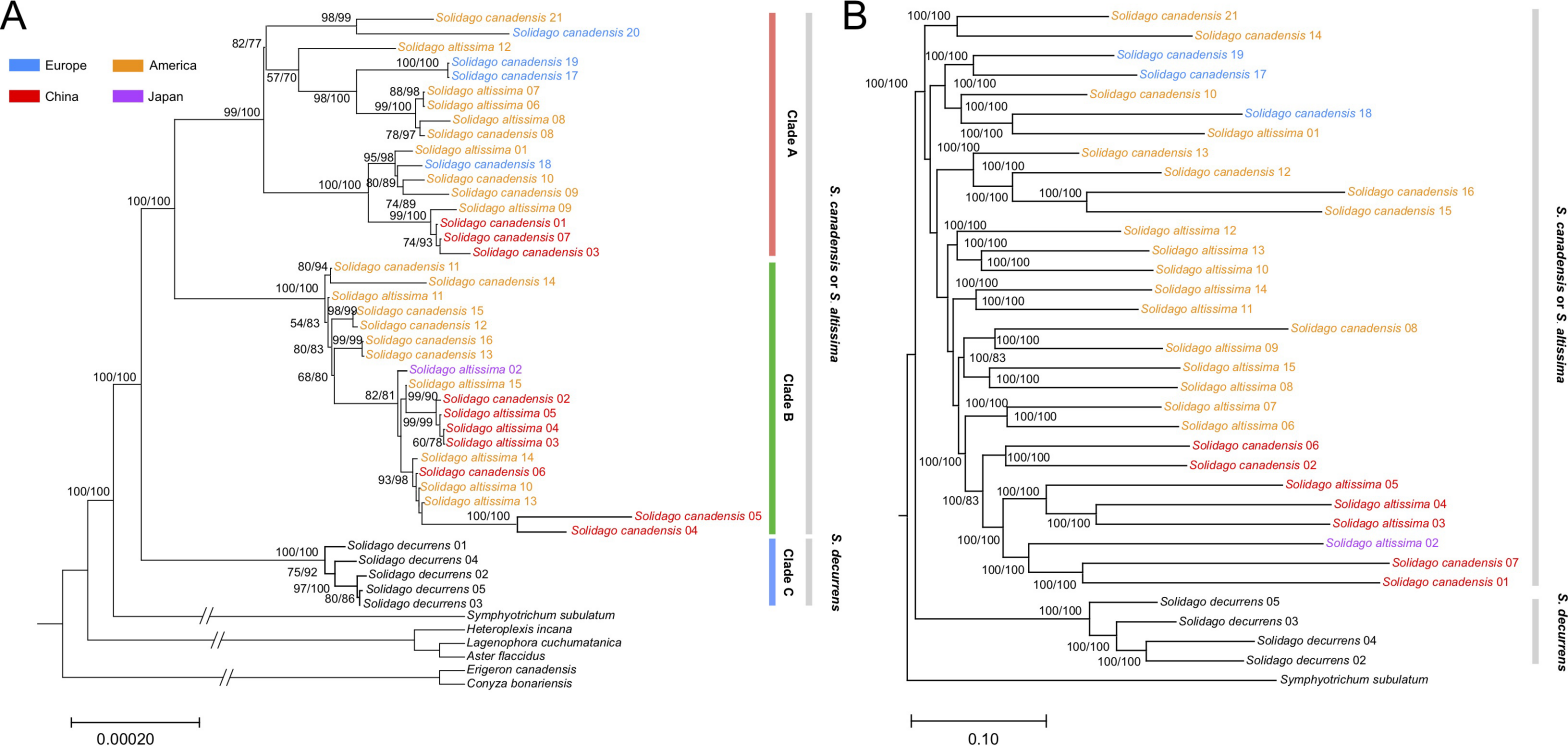
Maximum likelihood tree of collected individuals of *Solidago canadensis*, *S. altissima*, and *S. decurrens*. **(A)** Maximum likelihood tree constructed based on chloroplast genomes. Six published chloroplast genomes in Tribe. Astereae Cass were used as outgroups, including *Symphyotrichum subulatum*, *Aster flaccidus*, *Lagenophora cuchumatanica*, *Heteroplexis incana*, *Conyza bonariensis*, and *Erigeron canadensis*. **(B)** The maximum likelihood tree constructed based on nuclear genome-wide SNP data. *Symphyotrichum subulatum* was used as the outgroup. Due to missing nuclear genome data, the accessions *Solidago canadensis* 03, 04, 05, 09, 11, and 20 were not included in this analysis. A bootstrap value above 70% is labeled beside each node, numbers in parentheses are SH-aLRT support (%)/ultrafast bootstrap support (%). Individuals of the *Solidago canadensis* L. complex sampled in North America are colored orange, those from Europe blue, the one from Japan purple, and those from China red.

**Figure 3:**
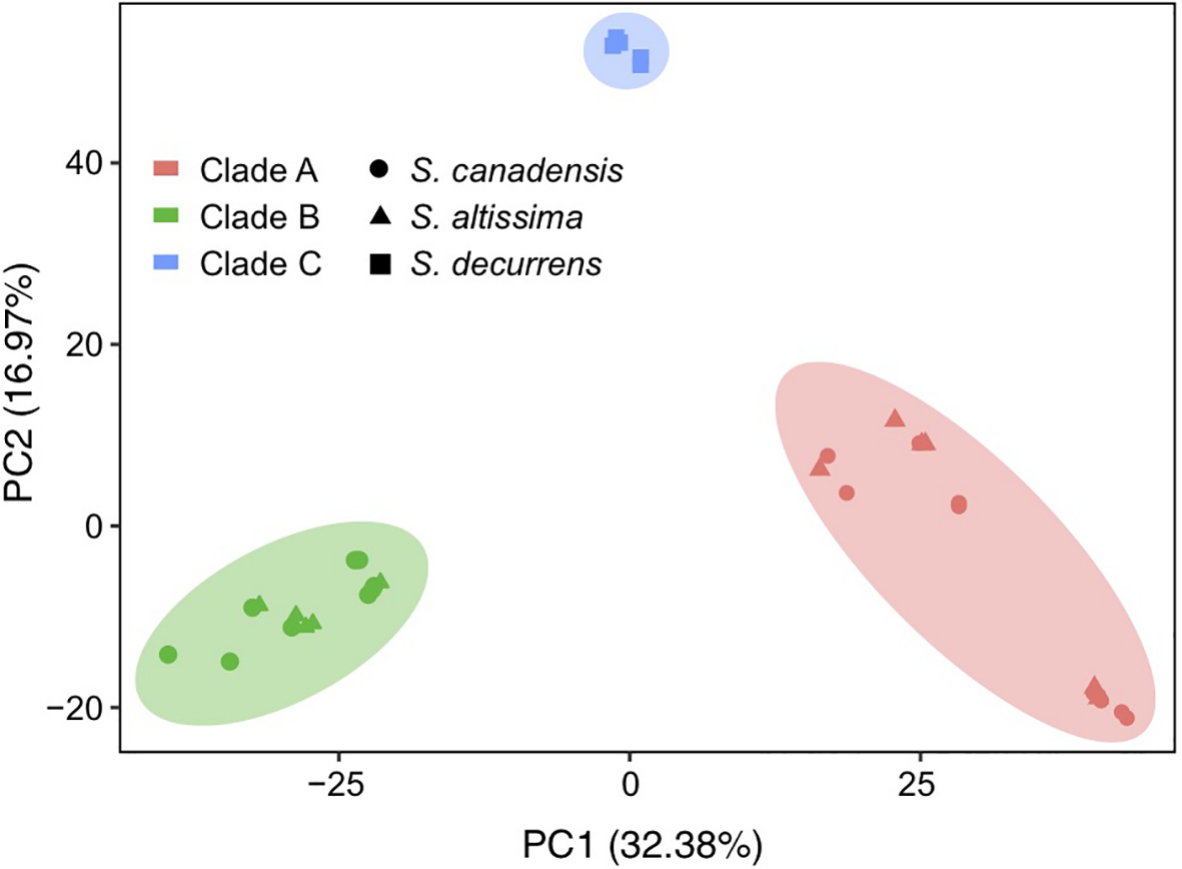
Principal component analysis of all chloroplast genomes based on SNPs. The chloroplast genomes of *S. canadensis* are represented as circles, those of *S. altissima* as triangles, and those of *S. decurrens* as squares. The chloroplast genomes located in clade A of the phylogenetic tree in **Figure 2** are colored red, those in clade B are colored green, and those in clade C are colored blue.

The topology of the phylogenetic trees based on the complete chloroplast genome sequences and nuclear genome-wide SNP data both had strong support (100% bootstrap value) and showed that all *S. canadensis* and *S. altissima* samples formed a monophyletic group, distinct from *S. decurrens* (**Figure 2**). Principal component analysis (PCA) based on the chloroplast genome sequences also significantly distinguished *S. decurrens* from *S. canadensis* and *S. altissima*, while *S. canadensis* and *S. altissima* were grouped together (**Figure 3**). These results suggest that *S. canadensis* and *S. altissima* are one and the same species or belong to one species complex.

Generally, the phylogenetic trees reconstructed from the chloroplast genome data and nuclear genome data showed a largely congruent invasion history of native and invasive *S. canadensis* L. complex (**Figure 2**). In the monophyletic group, individuals of the *S. canadensis* L. complex in China showed a close relationship with individuals from North America. In addition, one Japanese individual showed close affinities to the Chinese individuals. The European individuals were grouped together and also showed a close relationship with North American individuals. Taken together, the results of the phylogenetic analysis indicate multiple origins and complex invasive routes of the *S. canadensis* L. complex into China.

### 3.3 Comparative analyses of chloroplast genomes

To investigate the divergence of chloroplast genomes among the accessions of *S. altissima*, *S. canadensis*, and *S. decurrens*, multiple alignments of chloroplast genomes were compared by mVISTA, with the annotated accession *S. canadensis* 21 as reference (**Supplementary Figure S2**). The results revealed a high similarity of chloroplast genome sequences, but the LSC (from *trnH*-*GUG* to *rps19*) and *SSC* (from *ycf1* to *ndhF*) regions showed relatively less conservation compared to the IRA (from *ycf1* to *rpl2*) and IRB (from *rps19* to *ycf1*) regions (**Supplementary Figure S2**). Moreover, higher divergence was also observed in non-coding regions when compared to coding regions.

**Figure 4:**
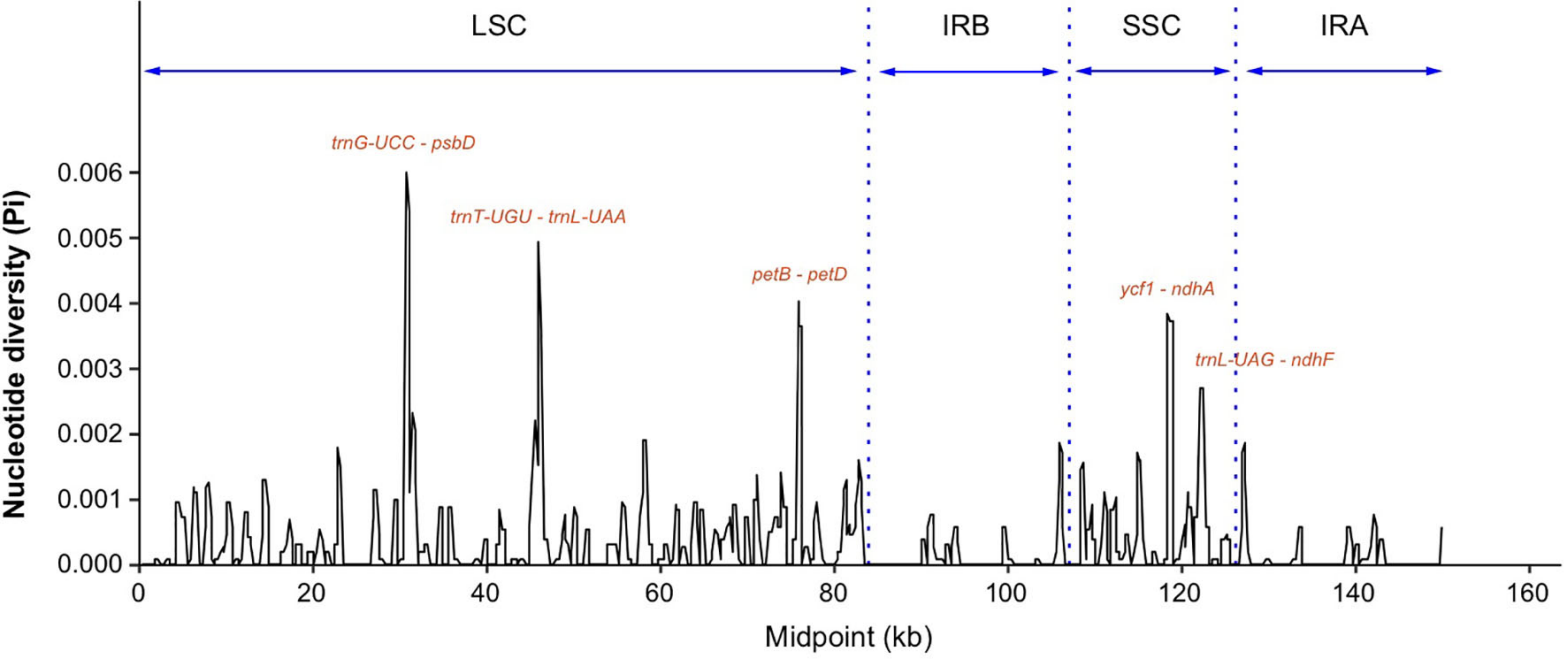
Sequence divergence analysis of chloroplast genomes of the *Solidago canadensis* L. complex and *S. decurrens*. Sliding window analysis of nucleotide diversity (Pi) had a window length of 600 bp and a step size of 200 bp. Each highly polymorphic region labeled by gene name was annotated on the graph. LSC, large single-copy region; SSC, small single-copy region; IRA and IRB were short for two inverted repeats. *psbD*, Photosystem II reaction center protein D; *petB*, Photosynthetic electron transfer; *petD*, Photosynthetic electron transfer D; *ycf1*, Hypothetical chloroplast reading frame; *ndhA*, NADP dehydrogenase subunit A; *ndhF*, NADP dehydrogenase subunit F; *trnG*-UCC, Transfer RNA glycine with UCC codon; *trnT*-UGU, Transfer RNA threonine with UGU codon; *trnL*-UAA, Transfer RNA leucine with UAA codon; *trnL*-UAG, Transfer RNA leucine with UAG codon.

Nucleotide diversity (Pi) values were calculated within 600 bp windows to discover hotspots of sequence divergence. The Pi values of the whole chloroplast genomes varied from 0 to 0.00594, and there was a higher diversity in the LSC and SSC regions than in the IR regions (**Figure 4**). A total of five highly variable regions with Pi values higher than 0.002 were identified, including *trnG*-*UCC* - *psbD*, *trnT*-*UGU* – *trnL*-*UAA* and *petB* - *petD* in the LSC region, and *ycf1* - *ndhA* and *trnL*-*UAG*-*ndhF* in the SSC region (**Figure 4**). The nucleotide diversity of individuals of the *S. canadensis* L. complex between China and Europe was higher than between individuals from China and North America (**Supplementary Figure S3**), which may be due to a limited sample size for Europe.

The non-synonymous (dN) and synonymous (dS) substitution rates of all the protein coding genes were analyzed across chloroplast genomes in the *S. canadensis* L. complex and *S. decurrens*. Most of the genes showed evidence of purifying selection. A total of 10 protein coding genes with significant posterior probabilities suggest that some sites of these genes were under positive selection (**Supplementary Table S4**). The gene *ycf1* showed the highest number (n = 8) of amino acid sites with evidence for positive selection (**Supplementary Table S4**). Interestingly, in the gene *rps19*, the 46th amino acid was alanine in the *S. altissima* and *S. canadensis* accessions, while it was serine in the *S. decurrens* accessions (**Supplementary Figure S4**).

## 4 Discussion

In this study, we found that the genome size (152,412-153,170 bp), GC content (37.16-37.29%), the quadripartite structure and gene composition (**Supplementary Table S2**) of the 40 *Solidago* accessions were consistent with those of other Tribe. Astereae Cass chloroplast genomes, and showed a highly conserved structure with minor differences among the *Solidago* species. The chloroplast genome features of the sampled *S. canadensis* L. complex and *S. decurrens* plants were largely identical (**Table 1** and **Supplementary Tables S2**, **S3**). Our results confirm the results of Tian et al. (2023), who used both nuclear molecular markers and morphological traits, that *S. decurrens* is different from the other accessions (Tian et al., 2023). More importantly, our whole chloroplast genome sequences revealed that there were no differences between the putative *S. canadensis* and *S. altissima* accessions, and between their native and non-native origins (**Figures 2** and **3**).

*Solidago canadensis* has spread worldwide owing to its high competitive ability, which allows it to dominate plant communities (Likhanov et al., 2021). *Solidago altissima* has also been reported as invasive in different parts of the world (Weber and Schmid, 1998). The taxonomic status of *S. canadensis* and *S. altissima* is still controversial, as it is not clear yet whether they are two separate species or belong to a single species or species complex (Croat, 1972; Weber, 2000; Semple et al., 2015). The main morphological traits that have been used to distinguish *S. canadensis* and *S. altissima* are the height and width of inflorescences (Croat, 1972), the numbers of disk and ray florets (Weber, 1997), pollen size (Melville and Morton, 1982) and the length and arrangement of the epidermal hairs of the stems and leaves (Szymura and Szymura, 2016). However, some studies showed that *S. canadensis* and *S. altissima* do not differ in functional traits, biomass production and allocation (Szymura and Szymura, 2013, 2015; Szymura et al., 2015). In addition, Tian et al. (2023) based on both molecular methods and morphological characteristics concluded that *S. canadensis* and *S. altissima* should be considered as a single species complex (Tian et al., 2023). As pointed out by others, next generation sequencing may help to reconstruct the phylogenetic relationships of *Solidago* species (Peirson et al., 2013; Shota et al., 2018; Semple et al., 2023). Indeed sequence data of chloroplast genomes and nuclear genomes have served as the technology of choice for phylogenetic investigations (Peirson et al., 2013; Guo et al., 2021). Similar to what earlier studies implied (Tian et al., 2023), our analyses of whole-chloroplast genome sequences and genome-wide SNP data (**Figures 2** and **3**) showed *S. canadensis* and *S. altissima* formed one monophyletic group as sister to *S. decurrens*. This supports the conclusion that *S. canadensis* and *S. altissima* belong to one species complex (i.e. the *S. canadensis* L. complex).

Accessions from China appeared in two different clades and one of these clades also included the accession from Japan and Europe (**Figure 2**). This is in line with the idea that there have been multiple introduction events of *S. canadensis* from its native North America range into China. The morphological analysis of Tian et al. (2023) based on 11 phenotypic traits also indicated that both the Chinese and European individuals in their study showed close affinity with North America individuals of *S. canadensis* L. complex. The Chinese accessions may have been introduced directly from North America or they may have used Japan or Europe as stepping stones (Dong et al., 2006; Yang et al., 2011). In any case, the results indicate that the *S. canadensis* L. complex came to China via multiple routes, as has also been suggested by other studies (Huang and Guo, 2004; Lin et al., 2023). For Europe, historical records indicate that *S. canadensis* was introduced as an ornamental plant in the 17th century (Loudon, 1850). This was several centuries before the first plants were introduced to China (in the 1930s) (Xu et al., 2012; Zhao et al., 2015). Therefore, it is likely that the invasive accessions of *S. canadensis* in Europe came directly from North America and were not introduced via China or Japan. So, despite some limitations regarding the number of studied individuals, our results still provide new insights into the complicated invasion history of the *S. canadensis* L. complex in Europe and Asia.

DNA barcode markers, which are usually located in highly variable genome regions, are widely used for species identification. However, as some of the classical DNA barcodes are insufficient to distinguish between different *Solidago* species, it is crucial to find more highly variable genome regions that could be developed as potential barcode markers for identification of *Solidago* species. We found that most variable regions were located in the LSC and SSC regions, and that non-coding regions were more variable than coding regions (**Supplementary Figure S2**). This is a common phenomenon in the chloroplast genomes of most angiosperms (Li D.-M. et al., 2021). In this study, five highly variable chloroplast-genome regions that are located in the intergenic region of *S. canadensis* L. complex and *S. decurrens* were identified based on mVISTA and nucleotide diversity. These regions include *trnG*-*UCC* - *psbD*, *trnT*-*UGG* - *trnL*-*UAA*, *petB* - *petD*, *ycf1* - *ndhA*, and *trnL*-*UAG* - *ndhF* (**Figure 4**), and could serve as potential DNA barcodes for identification and phylogenetic analysis of *Solidago* species. The most divergent regions of *ycf1* - *ndhA*, *trnL*-UAG - *ndhF*, *trnT*-UGU - *trnL*-UAA, and *trnG*-UCC - *psbD* as shown in **Figure 4** were consistent with results of previous studies (Li C. et al., 2021; Xia et al., 2022), indicating that these regions indeed evolve rapidly in *Solidago*.

Recently, *S. canadensis* has been added to the updated list of 33 key invasive alien species in China (http://www.moa.gov.cn/govpublic/KJJYS/202211/t20221109_6415160.htm; accessed on 1 March 2023). This indicates that the management and effective control of this species remains of pressing concern in China. Our study adds further evidence (see also Lin et al., 2023; Dong et al., 2016) that there may have been multiple introduction events of *S. canadensis* into China, and possibly via multiple pathways. To prevent invasions by more *S. canadensis* accessions, either from North America or other invaded regions, border quarantine measures should be strengthened. This does not only apply to *S. canadensis* but also to other alien species that may adversely affect local biodiversity. In addition, chemical, mechanical, and biological control approaches should be integrated to minimize the harm of *S. canadensis* to the local ecological environment (Wu and Qiang, 2005; Ma and Qiang, 2007; Jiang et al., 2008). Furthermore, with the application of next generation sequencing, the use of species-specific markers will be a very useful fast and cheap molecular tool for early detection of new alien species or accessions of alien species that are already invasive (Ardura, 2019). The molecular markers detected by our study may help to establish a rapid and effective early warning monitoring system to promptly detect *S. canadensis* so that it can be eradicated before it becomes invasive.

Positive selection is considered to play an important role in adaptive evolution, while negative (purifying) selection is a ubiquitous evolutionary force responsible for conservation of the genome across long evolutionary timescales (Cvijovic et al., 2018; Moseley et al., 2018). To detect signs of adaptive evolution among species, the ratio of substitution rates at synonymous and non-synonymous sites is frequently used (Kryazhimskiy and Plotkin, 2008; Williams et al., 2020). For our *Solidago* accessions, we identified 10 genes with sites that showed signals of positive selection. Among them, *rpoB*, *rbcL*, *accD*, *psbB*, *rps3*, *rps19*, *ndhH*, *ndhD*, and *ccsA* genes have also been found in other plant species (Li C. et al., 2021; Wen et al., 2021; Tao et al., 2023). Moreover, these genes could be used for identification and phylogenetic research of *Solidago* species. The gene *ycf1* had numerous such sites, indicating that this gene may play a key role in adaptive evolution of *Solidago* (**Supplementary Figure S4** and **Supplementary Table S4**). Furthermore, the genes *rps3* and *rps19*, encoding ribosomal subunit proteins that are considered to be essential for chloroplast biogenesis and function, showed signs of positive selection. This suggests that the studied *Solidago* species may increase evolutionary adaptability by regulating encoding ribosomal subunit proteins in chloroplast genomes. In addition, the likelihood ratio test (LRT) results showed that the p-values of *rpoB*, *rps19*, and *ndhD* genes were below 0.05, especially for the *rpoB* (encoding the beta-subunit of RNA polymerase) and *ndhD* gene (encoding NADP dehydrogenase), corroborating that these sites in the *S. canadensis* L. complex species have been under positive selection. As an important modulator of photosynthetic electron transport, recent study has revealed that positive selection of the *ndhD* gene was fairly common in all the main lineages of land plants (Wen et al., 2021). Moreover, the positive selection site of the *ndhD* gene in five *S. canadensis* L. complex individuals from China (*Solidago canadensis* 04 and 06) or three from North America (*Solidago altissima* 10, 13, and 14) contain same amino acid, indicating the Chinese individuals of the *S. canadensis* 04 and 06 may be directly introduced from North America. For the *rpoB* gene, *Solidago canadensis* 17 and 19 collected from Europe showed same amino acid in the 569^th^ positive selection site, inferring the direct introduction route from North America to Europe. In general, positive selection would possibly contribute to various invasive environments for this introduced species. Therefore, positive selection of these chloroplast genes may promote environmental adaptation of *Solidago*, but unravelling details of the adaptation mechanisms will need further in-depth research.

## Data Availability

The datasets presented in this study can be found in online repositories. The names of the repository/repositories and accession number(s) can be found in the article/**Supplementary Material**.

## Supplementary material

Supplementary Figure 1
*Solidago canadensis* in the field **(A)**, an herbarium specimen of *S. altissima* **(B)**, and a potted plant of *S. decurrens* **(C)**.

Supplementary Figure 2 Sequence alignment of chloroplast genomes with accession *Solidago canadensis* 21 as a reference. The y-scale indicates sequence identity from 50% to 100%. Gray arrows indicate the positions and directions of each gene. Red indicates non-coding sequences (CNS); Purple indicates the exons of protein-coding genes (exon); lime green indicates tRNAs and rRNAs.

Supplementary Figure 3 Nucleotide diversity analysis between Chinese and European/North American individuals of the *S. canadensis* L. complex.

Supplementary Figure 4 Partial alignment of three of the 10 positively selected genes with posterior probabilities higher than 0.95. **(A–C)** Partial aligned amino acid sequences of the *rpoB*, *rps19*, and *ndhD* genes, respectively. The red blocks represent amino acids with a high posterior probability.
