# Supplementary figures and images for: Complete chloroplast genome data reveal the existence of the Solidago canadensis L. complex and its potential introduction pathways into China
Source: Front Plant Sci. 2024 Dec 20;15:1498543. doi: 10.3389/fpls.2024.1498543 (PMC11695338; doi:10.3389/fpls.2024.1498543)

A.

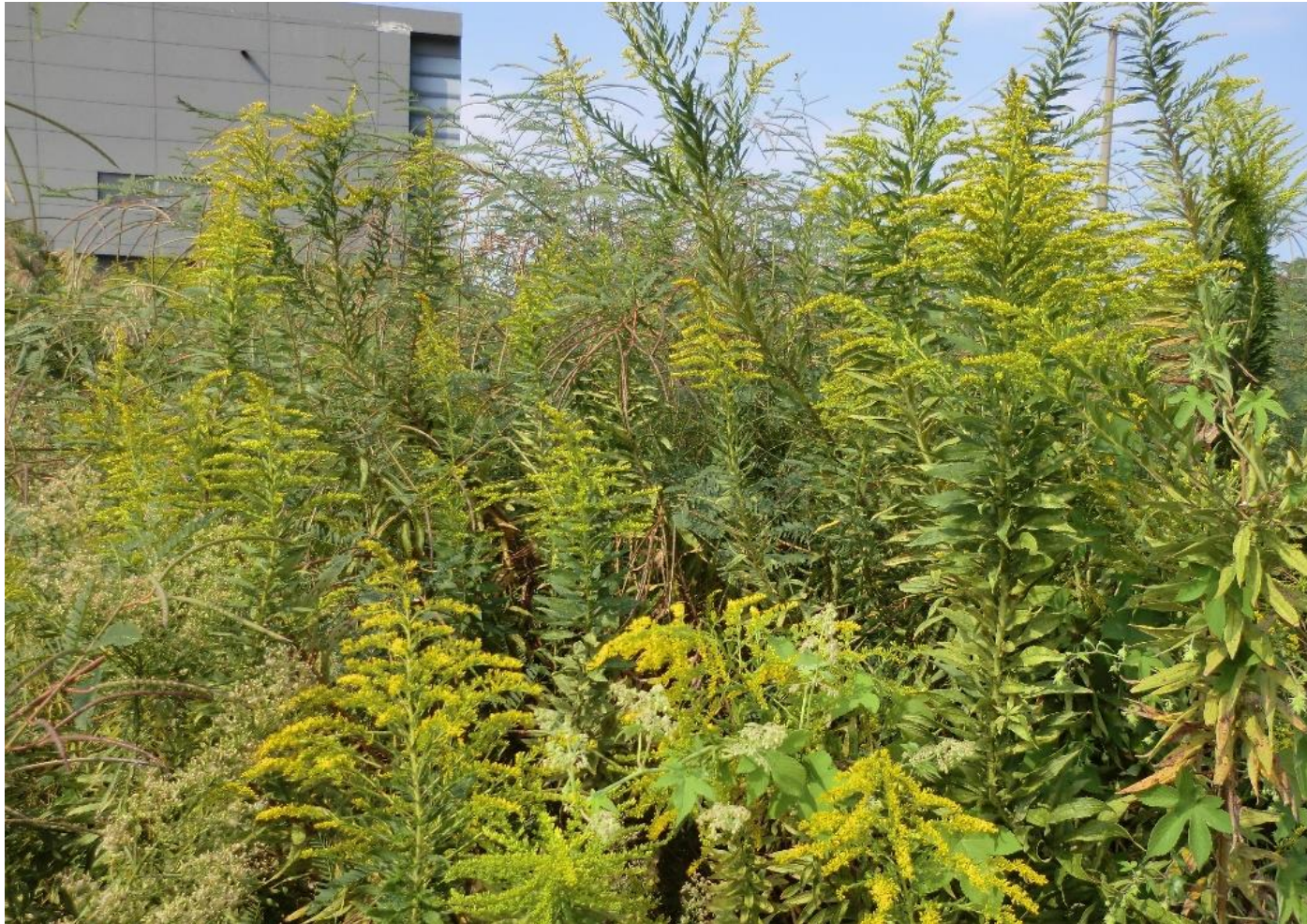

B.

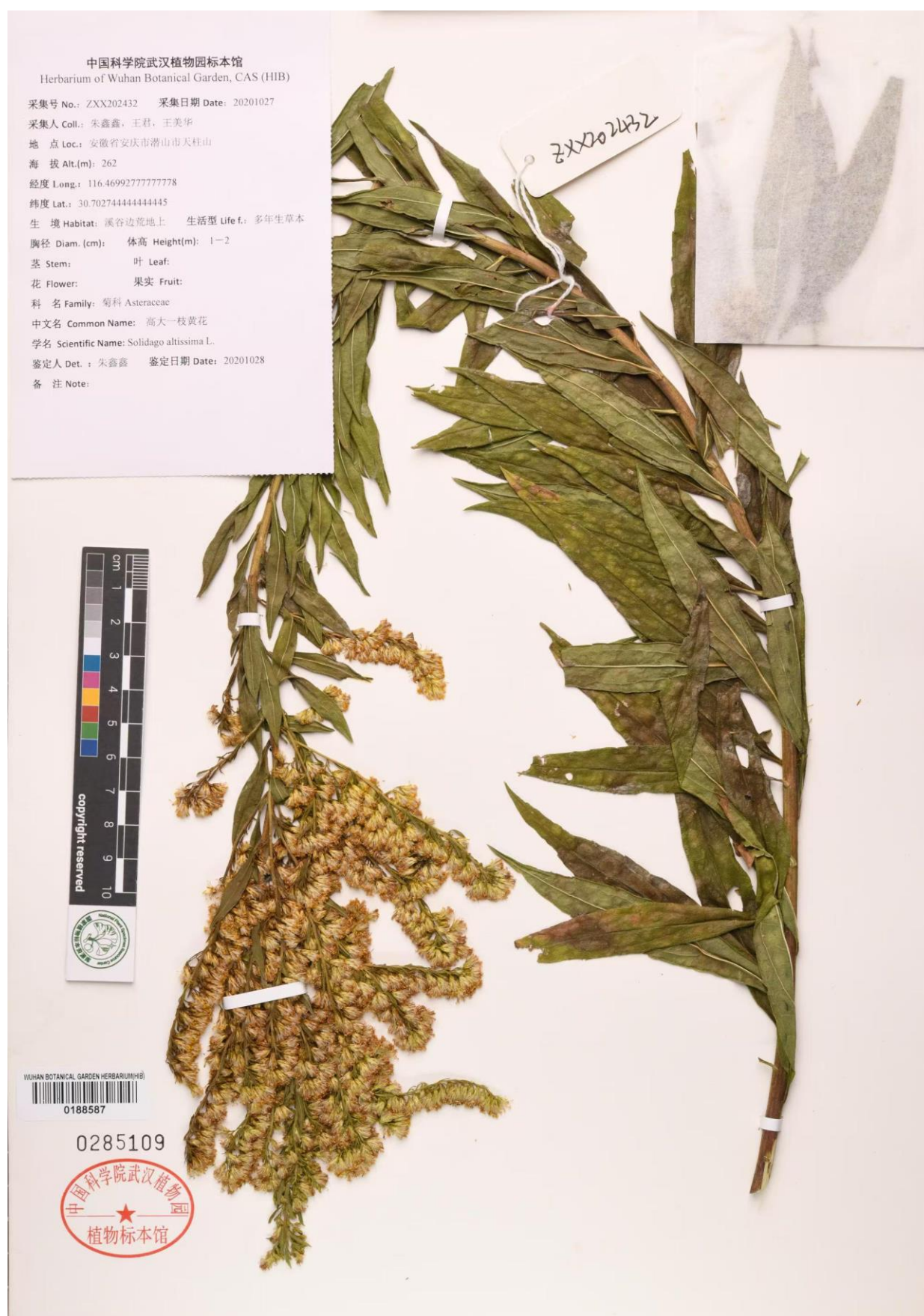

C.

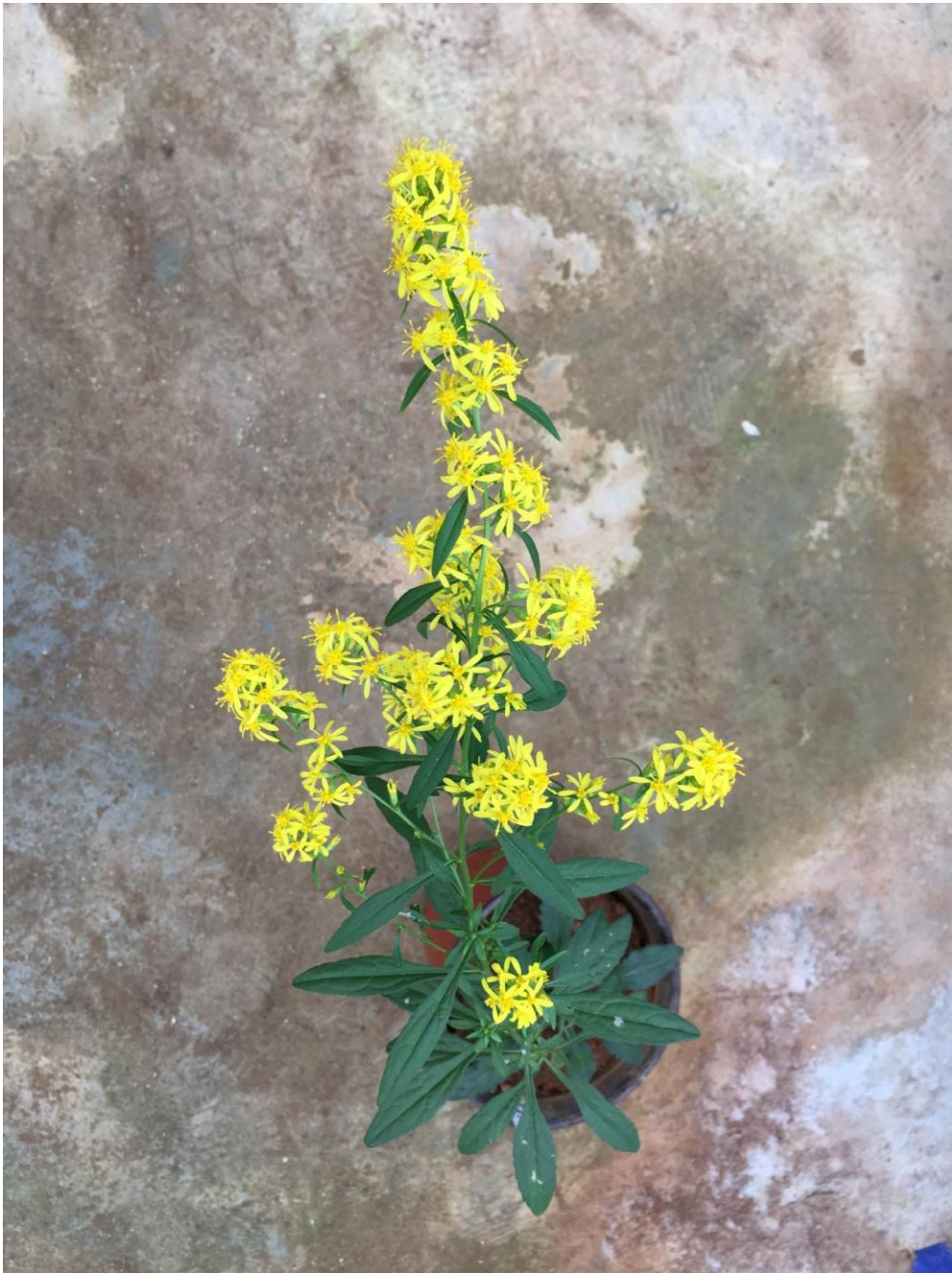

Supplement: Supplementary Figure 1 — Solidago canadensis in the field (A), an herbarium specimen of S. altissima (B), and a potted plant of S. decurrens (C). [file Image1.pdf]

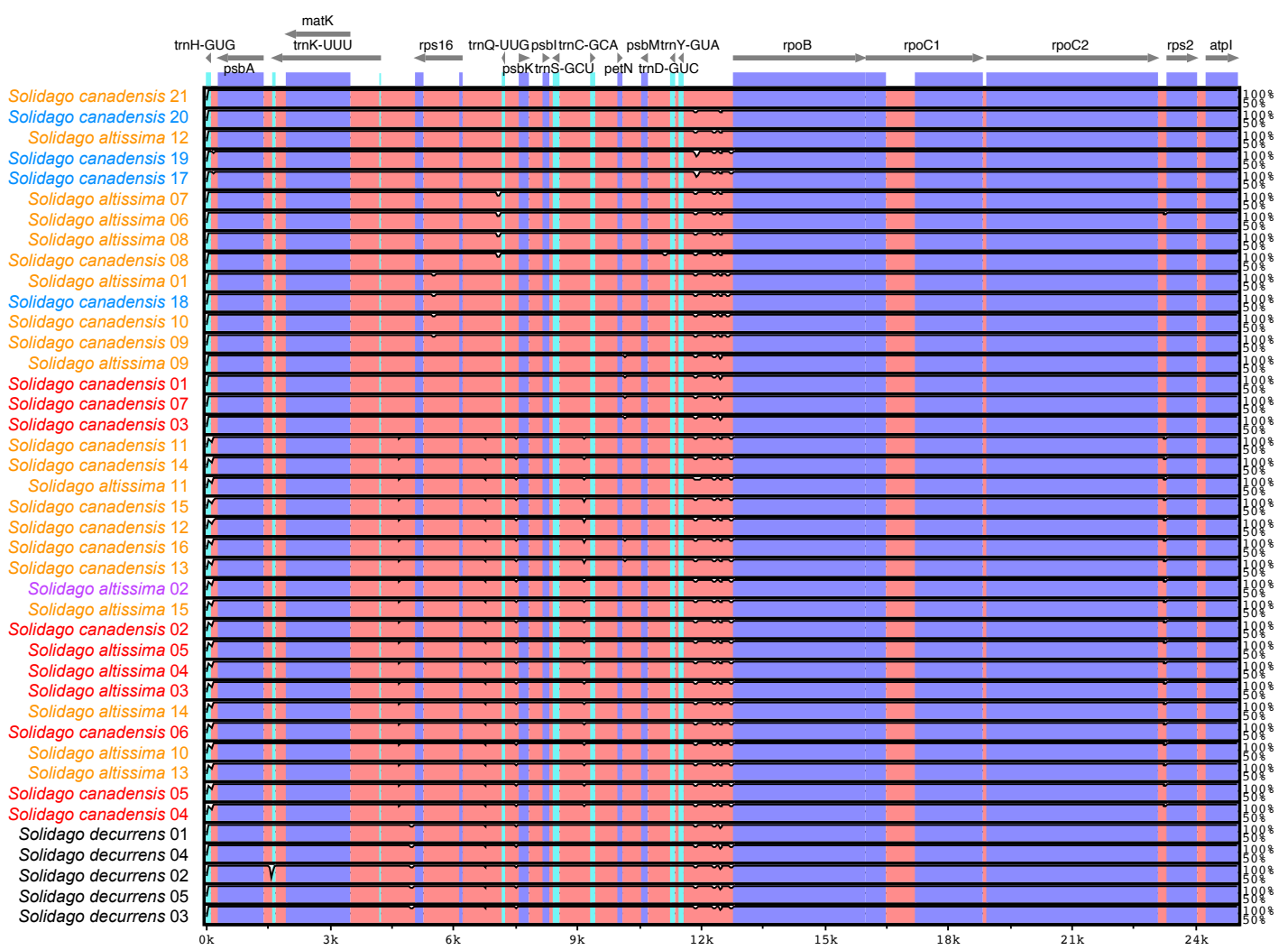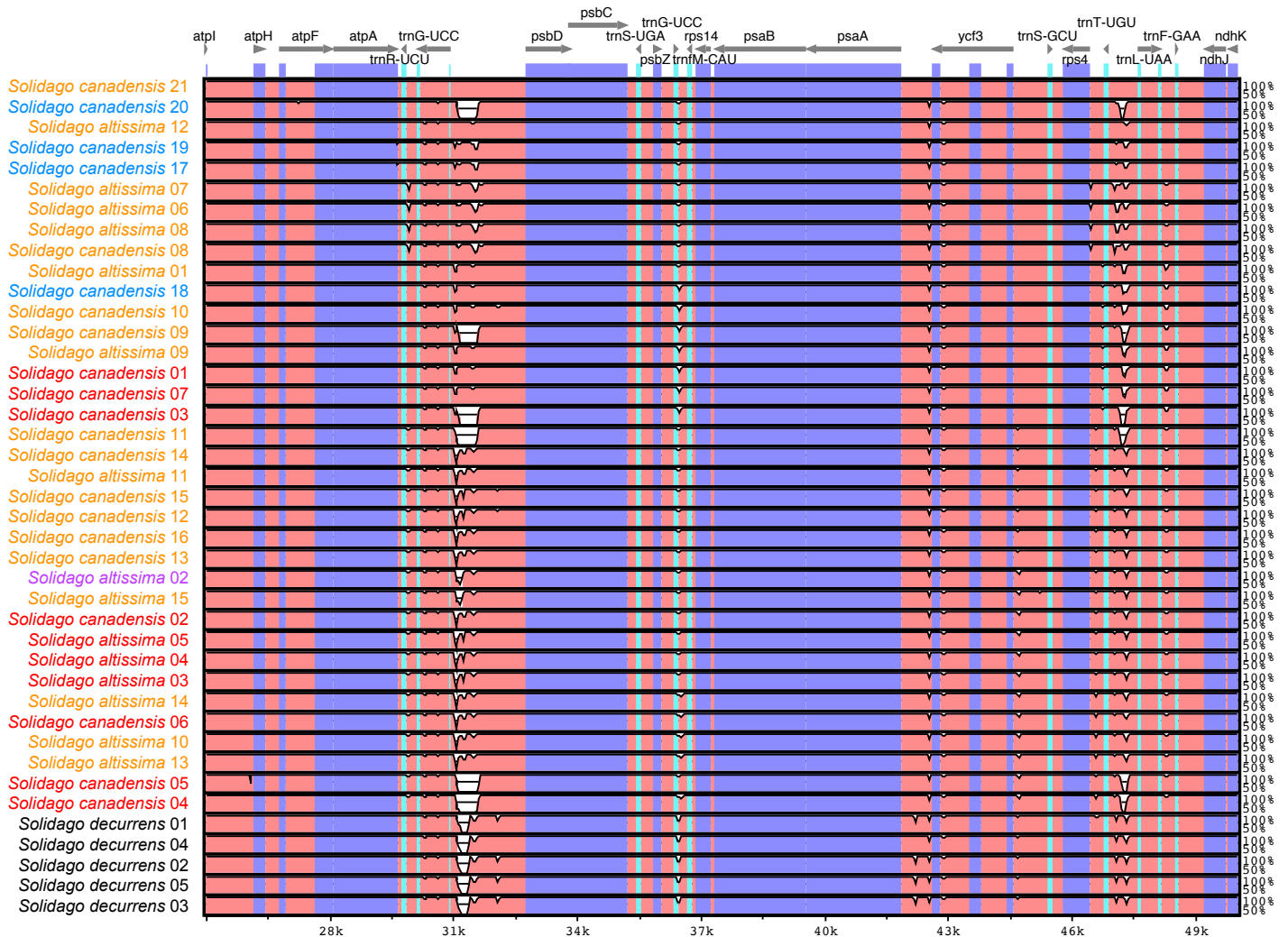

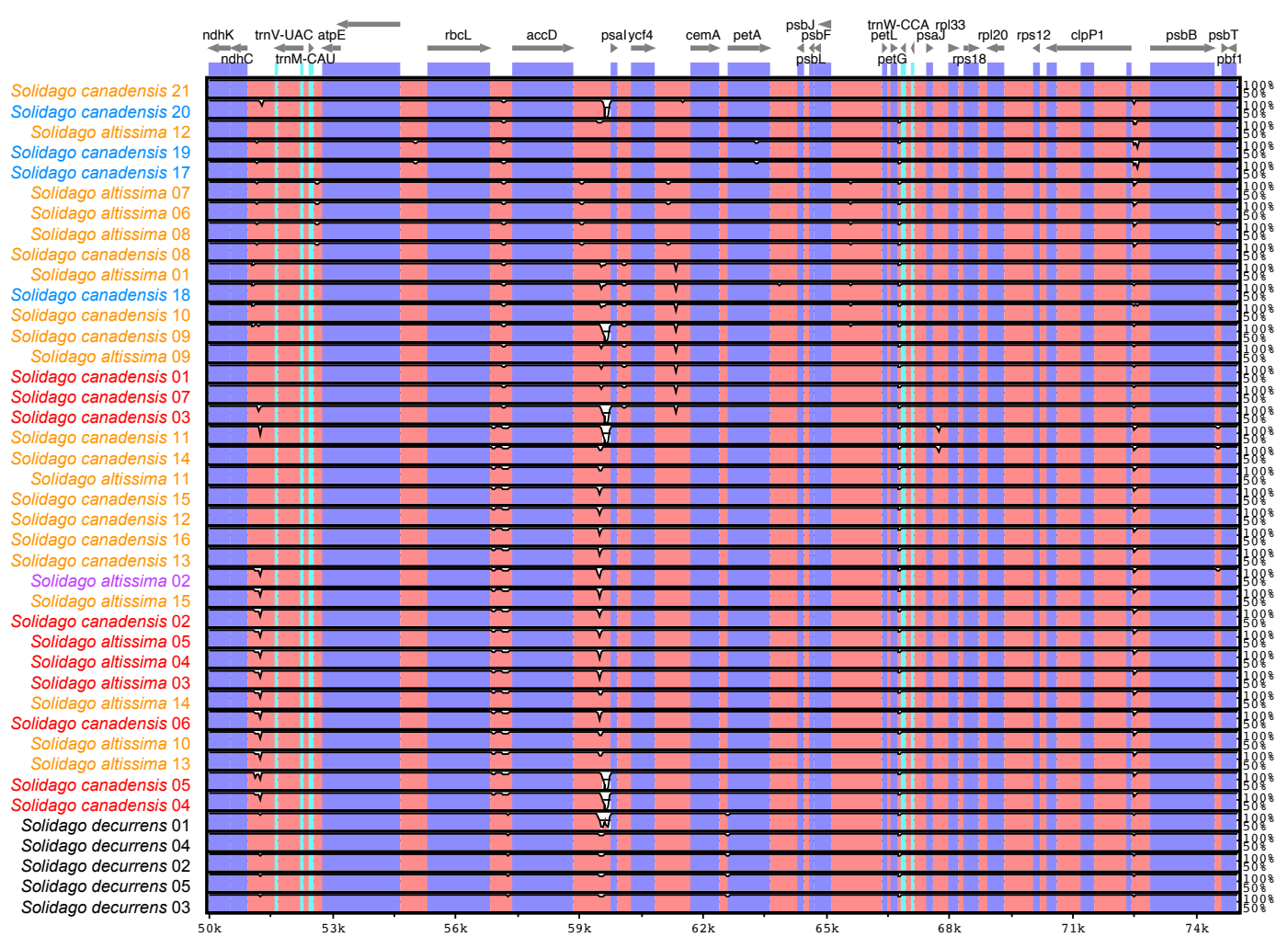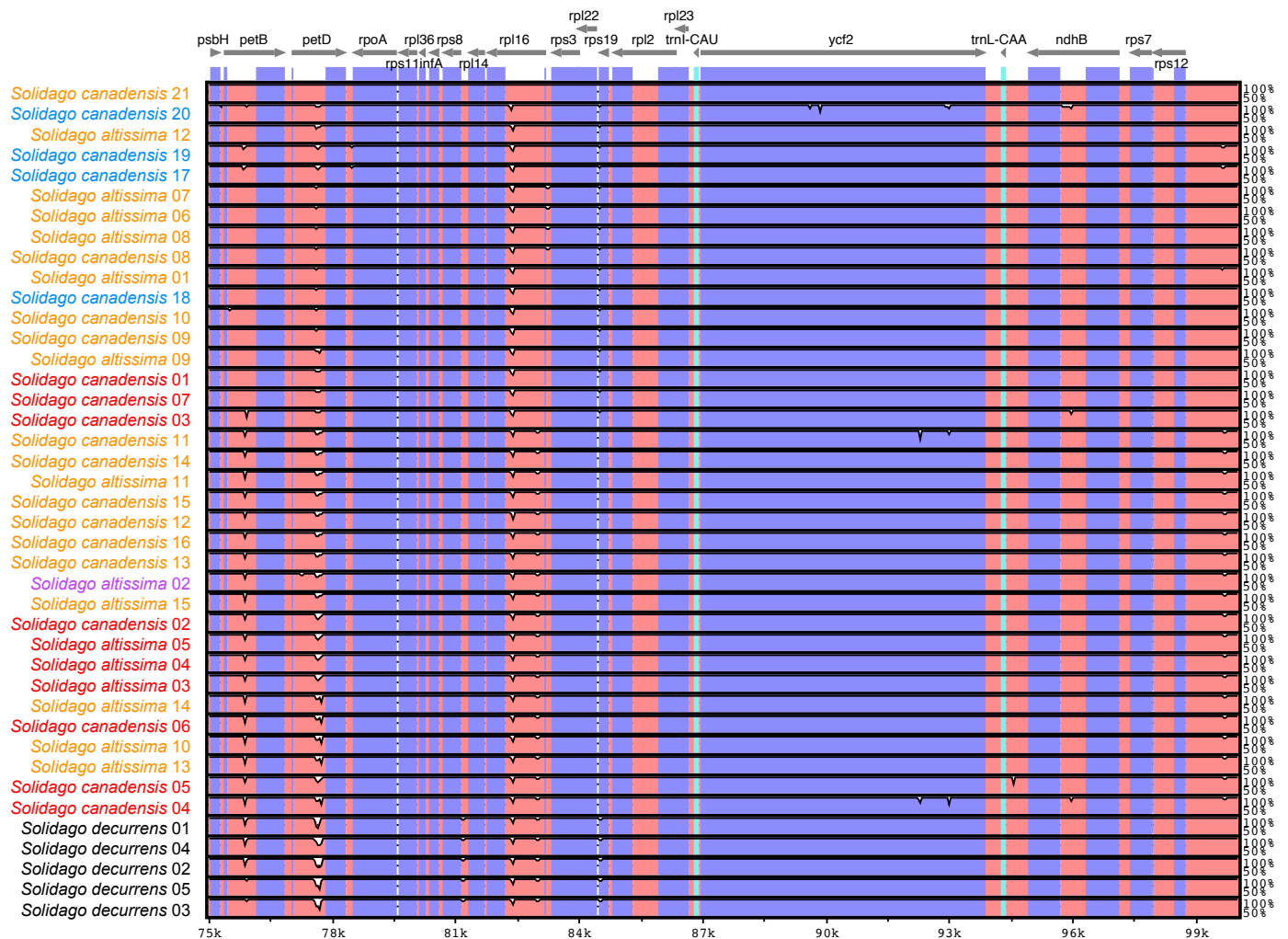

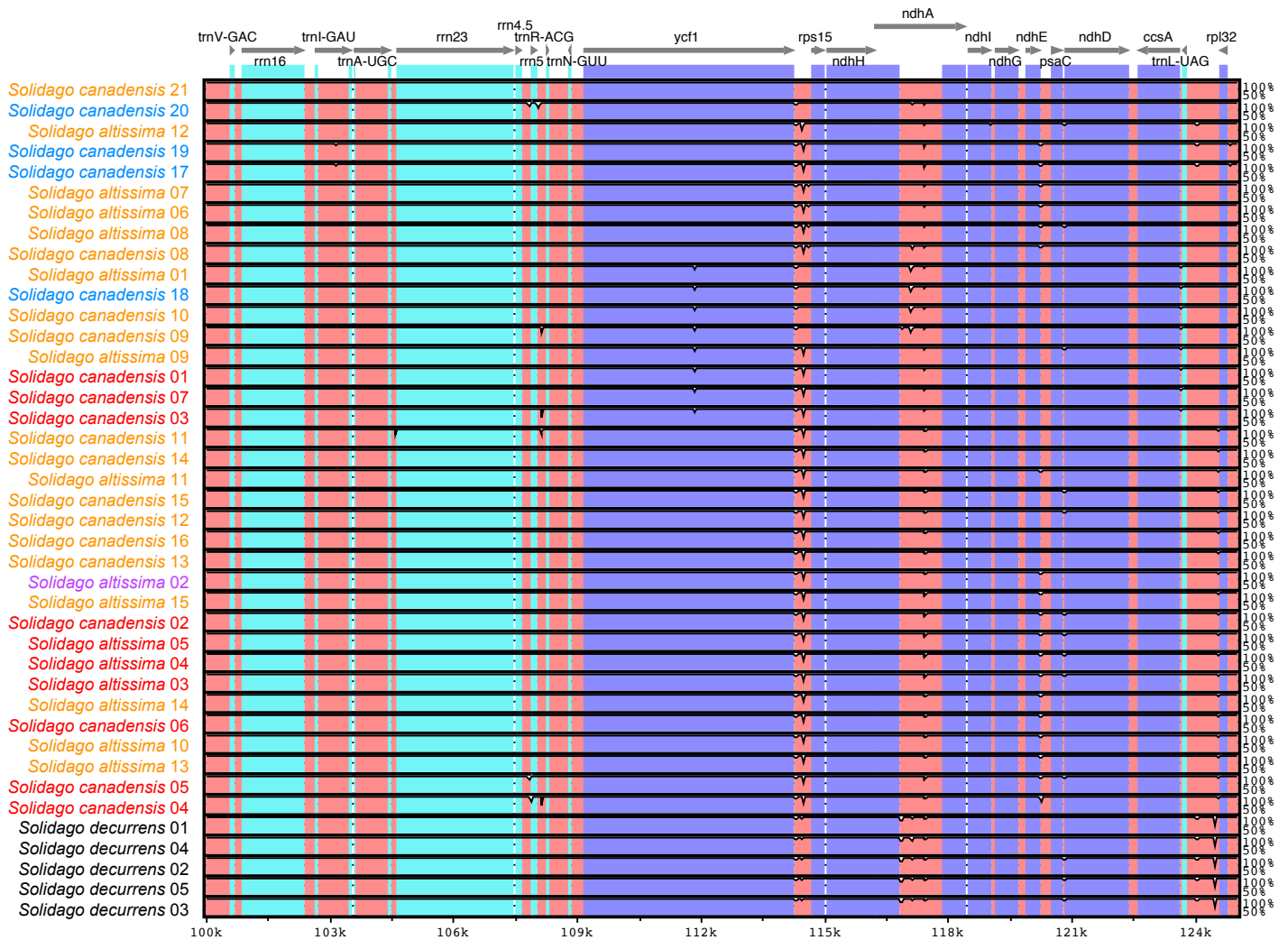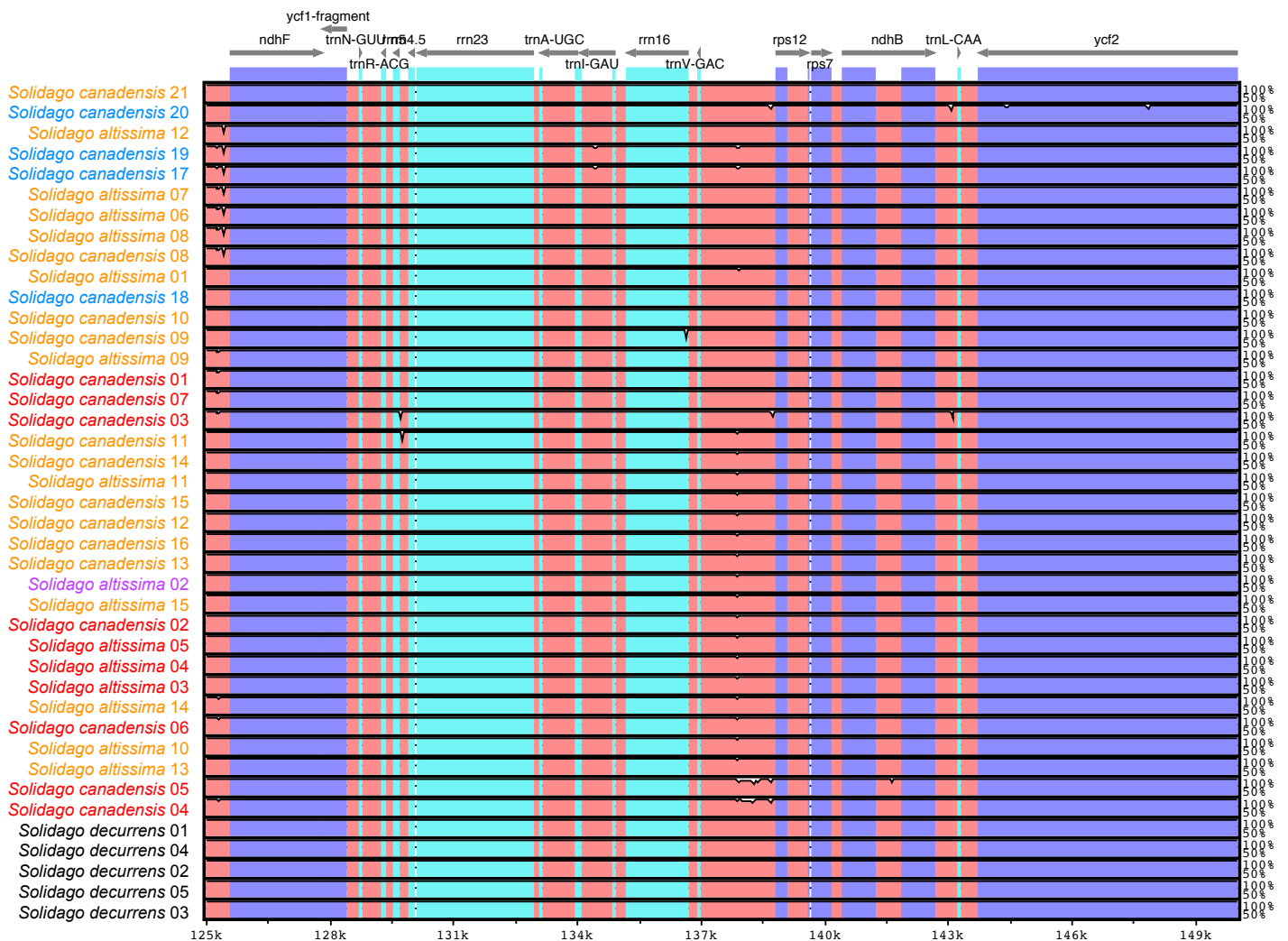

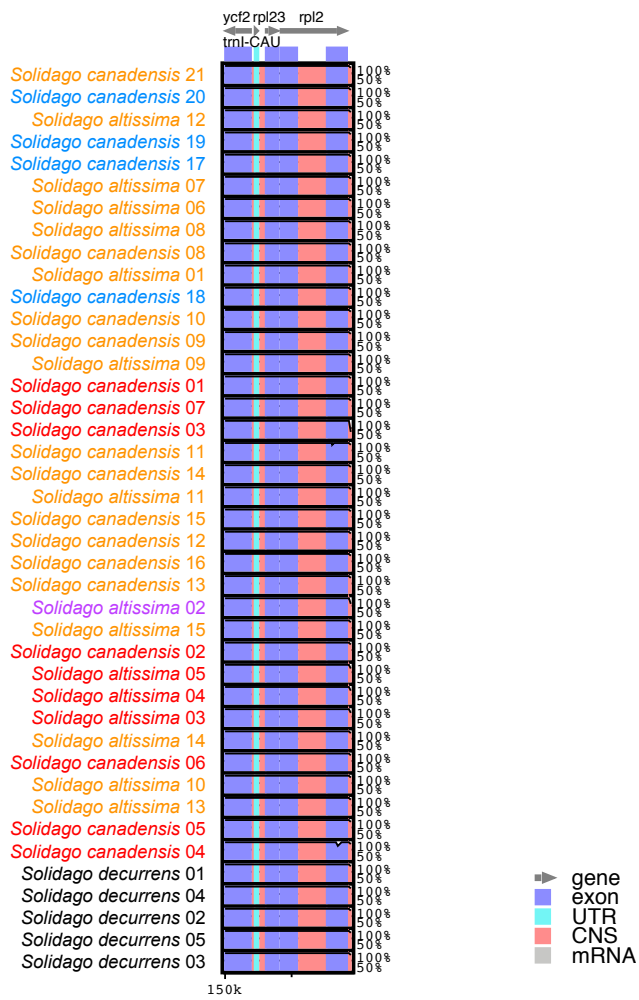

Supplement: Supplementary Figure 2 — Sequence alignment of chloroplast genomes with accession Solidago canadensis 21 as a reference. The y-scale indicates sequence identity from 50% to 100%. Gray arrows indicate the positions and directions of each gene. Red indicates non-coding sequences (CNS); Purple indicates the exons of protein-coding genes (exon); lime green indicates tRNAs and rRNAs. [file Image2.pdf]

Nucleotide diversity ( $P_i$ )

0.0015

0.0010

0.0005

0.0000

China vs Europe

China vs North America

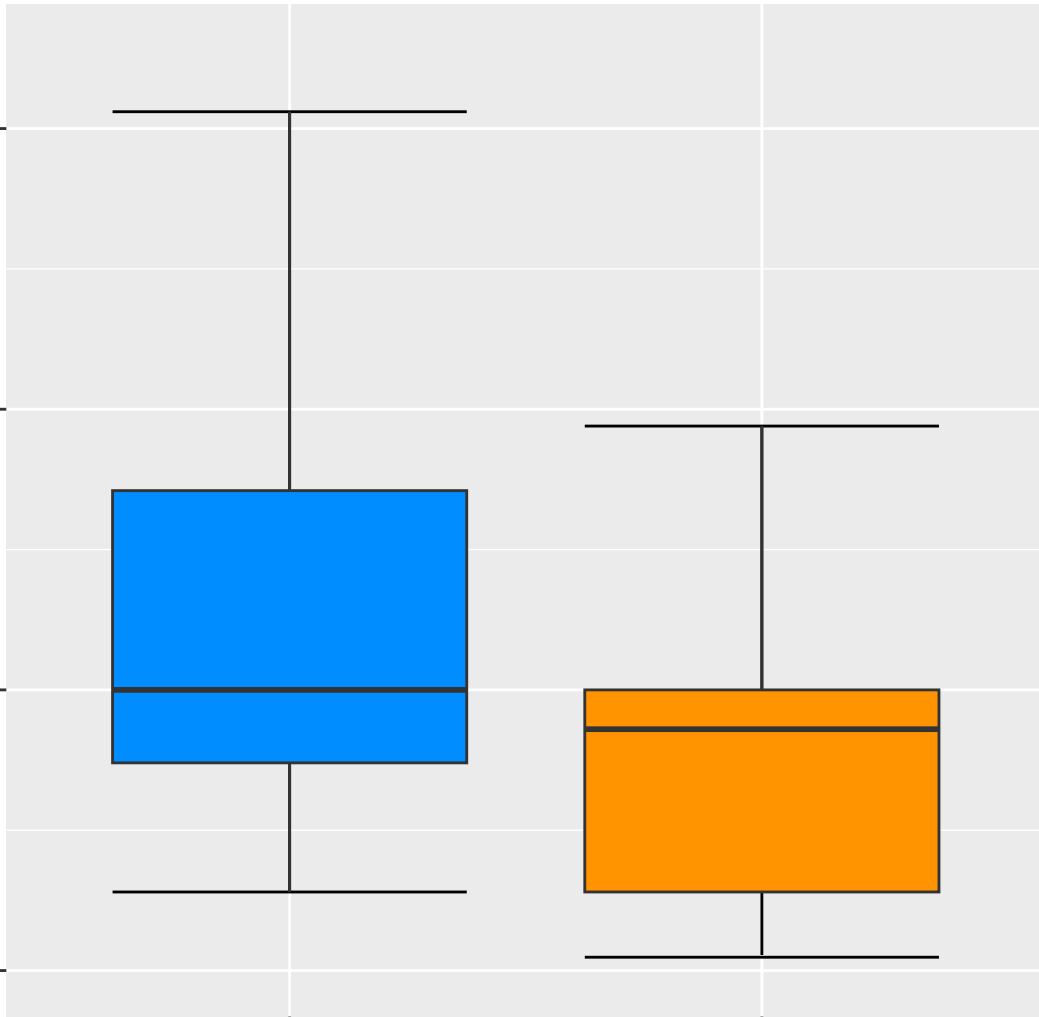

Supplement: Supplementary Figure 3 — Nucleotide diversity analysis between Chinese and European/North American individuals of the S. canadensis L. complex. [file Image3.pdf]

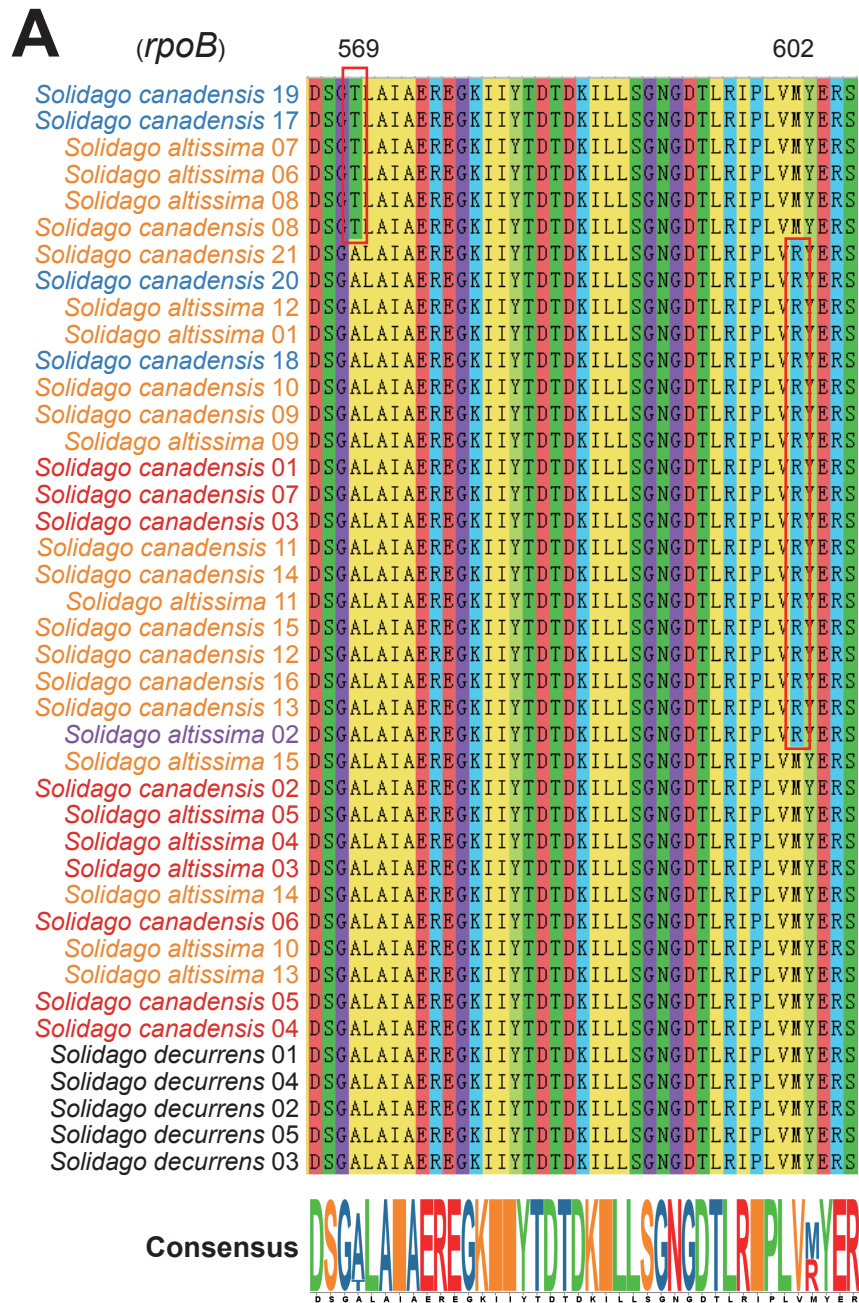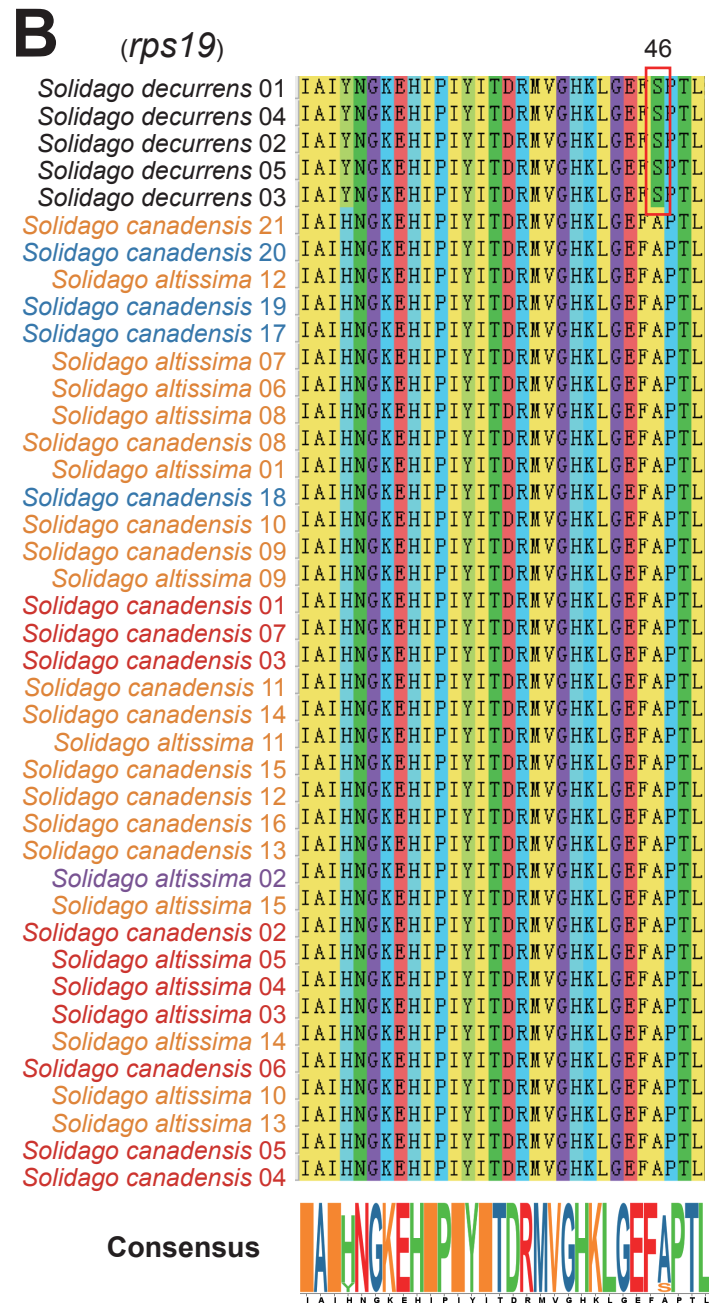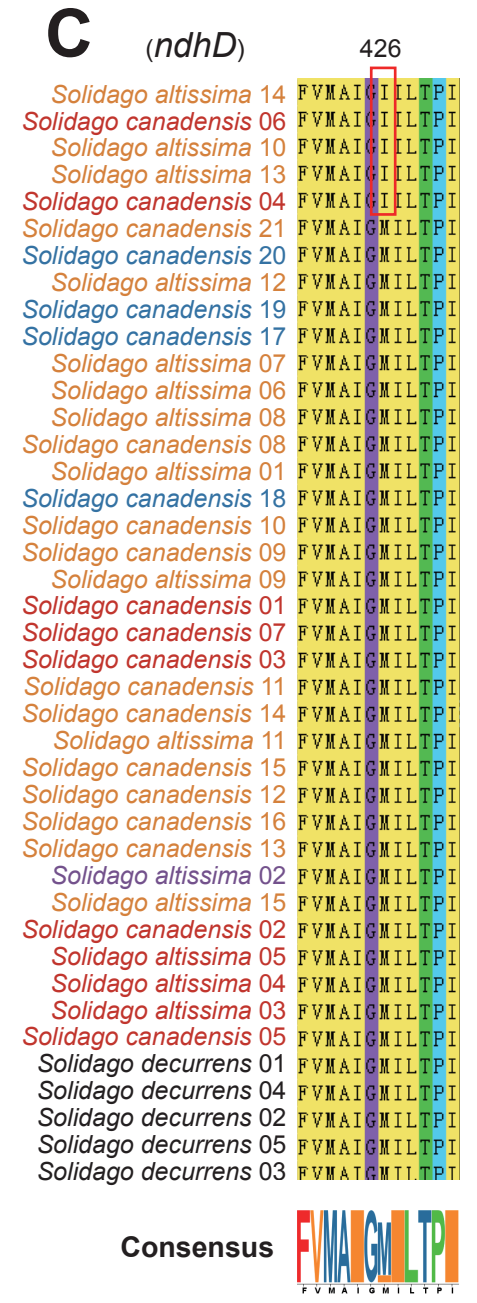

Supplement: Supplementary Figure 4 — Partial alignment of three of the 10 positively selected genes with posterior probabilities higher than 0.95. (A–C) Partial aligned amino acid sequences of the rpoB, rps19, and ndhD genes, respectively. The red blocks represent amino acids with a high posterior probability. [file Image4.pdf]
