# Supplementary material for: Complete chloroplast genome data reveal the existence of the Solidago canadensis L. complex and its potential introduction pathways into China
Source: Front Plant Sci. 2024 Dec 20;15:1498543. doi: 10.3389/fpls.2024.1498543 (PMC11695338; doi:10.3389/fpls.2024.1498543)
Supplement: Supplementary file 5 [file Table1.docx]

**Table S1. The plants identified as *Solidago canadensis*, *S. altissima* or *S. decurrens*, and of which the chloroplast genomes were sequenced in this study.**

| Species and number | Species | Region | Locality | Genbank accession | Specimen number | Material origin |
| --- | --- | --- | --- | --- | --- | --- |
| *Solidago canadensis* 01 | *Solidago canadensis* | China | Hangzhou, Zhejiang, China | OM273017 | HZU60102855 | herbarium material provided by Specimen Museum of Zhejiang University |
| *Solidago canadensis* 02 | *Solidago canadensis* | China | Nanjing, Jiangsu, China | OM273011 | - | field collected |
| *Solidago canadensis* 03 | *Solidago canadensis* | China | Nantong, Jiangsu, China | OM273007 | - | field collected |
| *Solidago canadensis* 04 | *Solidago canadensis* | China | Jiujiang, Jiangxi, China | OM272996 | - | field collected |
| *Solidago canadensis* 05 | *Solidago canadensis* | China | Pudong, Shanghai, China | OM272995 | - | field collected |
| *Solidago canadensis* 06 | *Solidago canadensis* | China | Hangzhou, Zhejiang, China | OM273014 | - | field collected |
| *Solidago canadensis* 07 | *Solidago canadensis* | China | Taizhou, Zhejiang, China | OM273000 | - | field collected |
| *Solidago canadensis* 08 | *Solidago canadensis* | North America | Maine, USA | OM273015 | - | field collected |
| *Solidago canadensis* 09 | *Solidago canadensis* | North America | Massachusetts, USA | OM273010 | - | field collected |
| *Solidago canadensis* 10 | *Solidago canadensis* | North America | Maine, USA | OM273001 | - | field collected |
| *Solidago canadensis* 11 | *Solidago canadensis* | North America | Maine, USA | OM273002 | - | field collected |
| *Solidago canadensis* 12 | *Solidago canadensis* | North America | Stewart park in Ithaca, New York, USA | OM272997 | - | field collected |
| *Solidago canadensis* 13 | *Solidago canadensis* | North America | Stewart park in Ithaca, New York, USA | OM273012 | - | field collected |
| *Solidago canadensis* 14 | *Solidago canadensis* | North America | John Day, Power River, USA | OM273013 | - | field collected |
| *Solidago canadensis* 15 | *Solidago canadensis* | North America | Stewart park in Ithaca, New York, USA | OM273004 | - | field collected |
| *Solidago canadensis* 16 | *Solidago canadensis* | North America | Stewart park in Ithaca, New York, USA | OM273016 | - | field collected |
| *Solidago canadensis* 17 | *Solidago canadensis* | Europe | Konstanz, Germany | OM272993 | - | field collected |
| *Solidago canadensis* 18 | *Solidago canadensis* | Europe | Potsdam, Germany | OM272998 | - | field collected |
| *Solidago canadensis* 19 | *Solidago canadensis* | Europe | Baden-Württemberg, Germany | OM273005 | - | field collected |
| *Solidago canadensis* 20 | *Solidago canadensis* | Europe | Welfingen, Switzerland | OM273006 | - | field collected |
| *Solidago canadensis* 21 | *Solidago canadensis* | North America | Oregon, USA | OM273009 | W6 52837 | grown from seed provided by GRIN/USDA |
| *Solidago altissima* 01 | *Solidago altissima* | North America | Massachusetts, USA | OR459629 | W6 57335 | grown from seed provided by GRIN/USDA |
| *Solidago altissima* 02 | *Solidago altissima* | Japan | Hata County, Kochi Prefecture, Japan | OR459630 | CSH0115776 | herbarium material provided by the Chinese Field Herbarium |
| *Solidago altissima* 03 | *Solidago altissima* | China | Anhui, China | OR459631 | HIB0188587 | herbarium material provided by the Wuhan Institute of Botany |
| *Solidago altissima* 04 | *Solidago altissima* | China | Anhui, China | OR459632 | HIB0188588 | herbarium material provided by the Wuhan Institute of Botany |
| *Solidago altissima* 05 | *Solidago altissima* | China | Anhui, China | OR459633 | HIB0188589 | herbarium material provided by the Wuhan Institute of Botany |
| *Solidago altissima* 06 | *Solidago altissima* | North America | Peterborough, Ontario, Canada | PP328569 | - | field collected |
| *Solidago altissima* 07 | *Solidago altissima* | North America | Peterborough, Ontario, Canada | PP328570 | - | field collected |
| *Solidago altissima* 08 | *Solidago altissima* | North America | Peterborough, Ontario, Canada | PP328571 | - | field collected |
| *Solidago altissima* 09 | *Solidago altissima* | North America | Peterborough, Ontario, Canada | PP328572 | - | field collected |
| *Solidago altissima* 10 | *Solidago altissima* | North America | Peterborough, Ontario, Canada | PP328573 | - | field collected |
| *Solidago altissima* 11 | *Solidago altissima* | North America | Peterborough, Ontario, Canada | PP328574 | - | field collected |
| *Solidago altissima* 12 | *Solidago altissima* | North America | Peterborough, Ontario, Canada | PP328575 | - | field collected |
| *Solidago altissima* 13 | *Solidago altissima* | North America | Peterborough, Ontario, Canada | PP328576 | - | field collected |
| *Solidago altissima* 14 | *Solidago altissima* | North America | Peterborough, Ontario, Canada | PP328577 | - | field collected |
| *Solidago altissima* 15 | *Solidago altissima* | North America | Peterborough, Ontario, Canada | PP328568 | - | field collected |
| *Solidago decurrens* 01 | *Solidago decurrens* | China | not available | NC_053705 | - | field collected |
| *Solidago decurrens* 02 | *Solidago decurrens* | China | Zhejiang, China | OM273008 | HZU60120963 | herbarium material provided by Specimen Museum of Zhejiang University |
| *Solidago decurrens* 03 | *Solidago decurrens* | China | Lishui, Zhejiang, China | OM273003 | - | field collected |
| *Solidago decurrens* 04 | *Solidago decurrens* | China | Linhai, Zhejiang, China | OM272999 | - | field collected |
| *Solidago decurrens* 05 | *Solidago decurrens* | China | Yunnan, China | OM272994 | - | field collected |
