# Supplementary material for: Complete chloroplast genome data reveal the existence of the Solidago canadensis L. complex and its potential introduction pathways into China
Source: Front Plant Sci. 2024 Dec 20;15:1498543. doi: 10.3389/fpls.2024.1498543 (PMC11695338; doi:10.3389/fpls.2024.1498543)
Supplement: Supplementary file 6 [file Table2.docx]

**Table S2. Chloroplast genome architecture of *Solidago* species that used in this study.**

| Genomes | Species | Genome length (bp) | GC content (%) | LSC length (bp) | SSC length (bp) | IR region (bp) | Gene  number | Protein-coding | tRNAs | rRNAs | No. of pseudo-genes |
| --- | --- | --- | --- | --- | --- | --- | --- | --- | --- | --- | --- |
| *Solidago canadensis* 01 | *S. canadensis* | 153,118 | 37.17 | 85,018 | 18,066 | 25,017 | 128 | 85 | 35 | 8 | 1 |
| *Solidago canadensis* 02 | *S. canadensis* | 152,960 | 37.20 | 84,828 | 18,084 | 25,024 | 128 | 85 | 35 | 8 | 1 |
| *Solidago canadensis* 03 | *S. canadensis* | 152,665 | 37.27 | 84,415 | 18,066 | 25,134 | 128 | 85 | 35 | 8 | 1 |
| *Solidago canadensis* 04 | *S. canadensis* | 152,500 | 37.27 | 84,214 | 18,104 | 25,029 | 128 | 85 | 35 | 8 | 1 |
| *Solidago canadensis* 05 | *S. canadensis* | 152,412 | 37.29 | 84,222 | 18,084 | 25,051 | 128 | 85 | 35 | 8 | 1 |
| *Solidago canadensis* 06 | *S. canadensis* | 153,057 | 37.18 | 84,914 | 18,095 | 25,024 | 128 | 85 | 35 | 8 | 1 |
| *Solidago canadensis* 07 | *S. canadensis* | 153,118 | 37.17 | 85,018 | 18,066 | 25017 | 128 | 85 | 35 | 8 | 1 |
| *Solidago canadensis* 08 | *S. canadensis* | 153,059 | 37.17 | 84,880 | 18,143 | 25,018 | 128 | 85 | 35 | 8 | 1 |
| *Solidago canadensis* 09 | *S. canadensis* | 152,539 | 37.27 | 84,332 | 18,076 | 25,060 | 128 | 85 | 35 | 8 | 1 |
| *Solidago canadensis* 10 | *S. canadensis* | 153,156 | 37.16 | 85,048 | 18,072 | 25,018 | 128 | 85 | 35 | 8 | 1 |
| *Solidago canadensis* 11 | *S. canadensis* | 152,529 | 37.28 | 84,242 | 18,096 | 25,060 | 128 | 85 | 35 | 8 | 1 |
| *Solidago canadensis* 12 | *S. canadensis* | 152,966 | 37.20 | 84,822 | 18,096 | 25,024 | 128 | 85 | 35 | 8 | 1 |
| *Solidago canadensis* 13 | *S. canadensis* | 152,956 | 37.20 | 84,811 | 18,097 | 25,024 | 128 | 85 | 35 | 8 | 1 |
| *Solidago canadensis* 14 | *S. canadensis* | 152,934 | 37.20 | 84,790 | 18,096 | 25,024 | 128 | 85 | 35 | 8 | 1 |
| *Solidago canadensis* 15 | *S. canadensis* | 152,956 | 37.20 | 84,813 | 18,095 | 25,024 | 128 | 85 | 35 | 8 | 1 |
| *Solidago canadensis* 16 | *S. canadensis* | 152,956 | 37.20 | 84,811 | 18,097 | 25,024 | 128 | 85 | 35 | 8 | 1 |
| *Solidago canadensis* 17 | *S. canadensis* | 153,097 | 37.17 | 84,930 | 18,137 | 25,015 | 128 | 85 | 35 | 8 | 1 |
| *Solidago canadensis* 18 | *S. canadensis* | 153,170 | 37.16 | 85,061 | 18,073 | 25,018 | 128 | 85 | 35 | 8 | 1 |
| *Solidago canadensis* 19 | *S. canadensis* | 153,097 | 37.17 | 84,930 | 18,137 | 25,015 | 128 | 85 | 35 | 8 | 1 |
| *Solidago canadensis* 20 | *S. canadensis* | 152,495 | 37.27 | 84,282 | 18,070 | 25,045 | 128 | 85 | 35 | 8 | 1 |
| *Solidago canadensis* 21 | *S. canadensis* | 152,828 | 37.21 | 84,719 | 18,075 | 25,017 | 128 | 85 | 35 | 8 | 1 |
| *Solidago altissima* 01 | *S. altissima* | 153,105 | 37.17 | 85,007 | 18,072 | 25,013 | 128 | 85 | 35 | 8 | 1 |
| *Solidago altissima* 02 | *S. altissima* | 152,980 | 37.20 | 84,863 | 18,089 | 25,014 | 128 | 85 | 35 | 8 | 1 |
| *Solidago altissima* 03 | *S. altissima* | 152,961 | 37.20 | 84,841 | 18,084 | 25,018 | 128 | 85 | 35 | 8 | 1 |
| *Solidago altissima* 04 | *S. altissima* | 152,961 | 37.20 | 84,841 | 18,084 | 25,018 | 128 | 85 | 35 | 8 | 1 |
| *Solidago altissima* 05 | *S. altissima* | 152,960 | 37.20 | 84,840 | 18,084 | 25,018 | 128 | 85 | 35 | 8 | 1 |
| *Solidago altissima* 06 | *S. altissima* | 153,033 | 37.18 | 84,863 | 18,134 | 25,018 | 128 | 85 | 35 | 8 | 1 |
| *Solidago altissima* 07 | *S. altissima* | 153,032 | 37.18 | 84,862 | 18,134 | 25,018 | 128 | 85 | 35 | 8 | 1 |
| *Solidago altissima* 08 | *S. altissima* | 153,031 | 37.18 | 84,860 | 18,135 | 25,018 | 128 | 85 | 35 | 8 | 1 |
| *Solidago altissima* 09 | *S. altissima* | 153,126 | 37.16 | 85,026 | 18,066 | 25,017 | 128 | 85 | 35 | 8 | 1 |
| *Solidago altissima* 10 | *S. altissima* | 153,019 | 37.19 | 84,882 | 18,089 | 25,024 | 128 | 85 | 35 | 8 | 1 |
| *Solidago altissima* 11 | *S. altissima* | 152,957 | 37.20 | 84,809 | 18,100 | 25,024 | 128 | 85 | 35 | 8 | 1 |
| *Solidago altissima* 12 | *S. altissima* | 153,068 | 37.17 | 84,867 | 18,165 | 25,018 | 128 | 85 | 35 | 8 | 1 |
| *Solidago altissima* 13 | *S. altissima* | 153,019 | 37.19 | 84,884 | 18,089 | 25,024 | 128 | 85 | 35 | 8 | 1 |
| *Solidago altissima* 14 | *S. altissima* | 153,025 | 37.19 | 84,882 | 18,095 | 25,024 | 128 | 85 | 35 | 8 | 1 |
| *Solidago altissima* 15 | *S. altissima* | 152,959 | 37.20 | 84,827 | 18,084 | 25,024 | 128 | 85 | 35 | 8 | 1 |
| *Solidago decurrens* 01 | *S. decurrens* | 152,728 | 37.22 | 84,599 | 18,089 | 25,020 | 128 | 85 | 35 | 8 | 1 |
| *Solidago decurrens* 02 | *S. decurrens* | 152,846 | 37.19 | 84,717 | 18,091 | 25,019 | 128 | 85 | 35 | 8 | 1 |
| *Solidago decurrens* 03 | *S. decurrens* | 152,863 | 37.19 | 84,736 | 18,089 | 25,019 | 128 | 85 | 35 | 8 | 1 |
| *Solidago decurrens* 04 | *S. decurrens* | 152,830 | 37.20 | 84,684 | 18,095 | 25,020 | 128 | 85 | 35 | 8 | 1 |
| *Solidago decurrens* 05 | *S. decurrens* | 152,863 | 37.19 | 84,736 | 18,089 | 25,019 | 128 | 85 | 35 | 8 | 1 |
