# Supplementary material for: Complete chloroplast genome data reveal the existence of the Solidago canadensis L. complex and its potential introduction pathways into China
Source: Front Plant Sci. 2024 Dec 20;15:1498543. doi: 10.3389/fpls.2024.1498543 (PMC11695338; doi:10.3389/fpls.2024.1498543)
Supplement: Supplementary file 7 [file Table3.docx]

**Table S3. Genes with introns in the chloroplast genomes.**

| Gene | Location | Exon I (bp) | Intron I (bp) | Exon II (bp) | Intron II (bp) | Exon III (bp) |
| --- | --- | --- | --- | --- | --- | --- |
| *trnK-UUU* | LSC | 37 | 2536 | 30 | - | - |
| *rps16* | LSC | 40 | 882 - 891 | 185 | - | - |
| *rpoC1* | LSC | 432 | 748 | 1638 | - | - |
| *atpF* | LSC | 145 | 712 - 717 | 410 | - | - |
| *trnG-UCC* | LSC | 23 | 733 - 741 | 47 | - | - |
| *ycf3* | LSC | 126 | 690 | 228 | 739 - 744 | 153 |
| *trnL-UAA* | LSC | 37 | 428 | 50 | - | - |
| *trnV-UAC* | LSC | 38 | 573 | 37 | - | - |
| *rps12* | LSC | 114 | - | 232 | 535 | 26 |
| *clpP1* | LSC | 71 | 811 - 812 | 292 | 615 - 616 | 228 |
| *petB* | LSC | 6 | 775 - 821 | 642 | - | - |
| *petD* | LSC | 8 | 820 - 852 | 475 | - | - |
| *rpl16* | LSC | 9 | 971 - 1222 | 399 | - | - |
| *rpl2* | IR | 390 | 671 | 435 | - | - |
| *ndhB* | IR | 777 | 673 - 682 | 756 | - | - |
| *trnI-GAU* | IR | 43 | 778 - 781 | 35 | - | - |
| *trnA-UGC* | IR | 38 | 819 | 35 | - | - |
| *ndhA* | SSC | 553 | 1093 - 1123 | 539 | - | - |
