# Supplementary material for: Complete chloroplast genome data reveal the existence of the Solidago canadensis L. complex and its potential introduction pathways into China
Source: Front Plant Sci. 2024 Dec 20;15:1498543. doi: 10.3389/fpls.2024.1498543 (PMC11695338; doi:10.3389/fpls.2024.1498543)
Supplement: Supplementary file 8 [file Table4.docx]

**Table S4. Positive selective amino acid loci and estimation of parameters.**

| Gene | Ln L | Estimates of parameters | Positively selected sites |
| --- | --- | --- | --- |
| *rpoB* | -4209.689106 | p0=0.00001 p=0.00500 q=2.33777  (p1=0.99999) ω=999.00000 | 569 A 0.962*, 602 R 0.962* |
| *rbcL* | -1943.621652 | p0=0.99380 p=0.00500 q=1.95204  (p1=0.00620) ω=60.66352 | 470 Q 0.541 |
| *accD* | -1866.854238 | p0=0.00001 p=2.05382 q=0.00500  (p1=0.99999) ω=999.00000 | 46 D 0.923 |
| *psbB* | -2034.517439 | p0=0.99999 p=60.74100 q=94.81534  (p1=0.00001) ω=169.41460 | 51 V 0.576 |
| *rps3* | -846.562444 | p0=0.00001 p=2.05354 q=0.00500  (p1=0.99999) ω=999.00000 | 104 N 0.923 |
| *rps19* | -263.371307 | p0=0.00001 p=99.00000 q=5.96041  (p1=0.99999) ω=999.00000 | 46 A 0.962* |
| *ycf1* | -6553.261067 | p0=0.98081 p=0.00500 q=5.67982  (p1=0.01919) ω=14.98847 | 429 K 0.559, 432 D 0.860, 875 L 0.561, 893 I 0.556, 1275 G 0.562, 1314 I 0.555, 1329 R 0.564, 1629 S 0.554 |
| *ndhH* | -1572.276508 | p0=0.99999 p=18.63435 q=57.92779  (p1=0.00001) ω=1.00000 | 157 Y 0.521 |
| *ndhD* | -1983.255461 | p0=0.00001 p=0.32733 q=0.00500  (p1=0.99999) ω=999.00000 | 426 M 0.962* |
| *ccsA* | -1245.180733 | p0=0.00001 p=2.05358 q=0.00500  (p1=0.99999) ω=999.00000 | 199 F 0.923 |
